# Apple crown and collar canker and necrosis caused by *Cytospora balanejica* sp. nov. in Iran

**DOI:** 10.1038/s41598-024-57235-3

**Published:** 2024-03-19

**Authors:** Razmig Azizi, Youbert Ghosta, Abdollah Ahmadpour

**Affiliations:** 1 Department of Plant Protection, Faculty of Agriculture, Urmia University, Urmia, Iran; 2 Higher Education Center of Shahid Bakeri, Urmia University, Miyandoab, Iran

**Keywords:** Plant sciences, Plant stress responses, Biotic

## Abstract

Apple is the most important fruit tree in West Azarbaijan province of Iran. In a survey of apple orchards, a disease with crown and collar canker and necrosis symptoms was observed in three young apple orchards in Urmia, affecting 15% and 1% of ‘Red Delicious’ and ‘Golden Delicious’ cultivars, respectively. A fungus with typical characteristics of the asexual morph of *Cytospora* was regularly isolated from the diseased tissues. Morphological characteristics and phylogenetic analyses inferred from the combined dataset of the ITS-rDNA, parts of LSU, *tef1-α*, *rpb2*, and *act1* genes revealed that the isolates represent a new species of *Cytospora*, described herein as *Cytospora balanejica* sp. nov.. The pathogenicity of all isolates was confirmed on apple cv. ‘Red Delicious’ based on Koch’s postulates. Also, the reaction of 12 other apple cultivars was assessed against five selected isolates with the highest virulence. The results showed that except for cv. ‘Braeburn’, which did not produce any symptoms of the disease, the other 11 cultivars showed characteristic disease symptoms including sunken and discolored bark and wood. The mean length of the discolored area was different among the 11 so-called susceptible cultivars, hence cvs. ‘M4’ and ‘Golden Delicious’ showed the highest and the lowest lesion length, respectively. Moreover, the aggressiveness of the five tested isolates was different, and the isolates BA 2-4 and BA 3-1 had the highest and lowest aggressiveness, respectively. Based on our observations on the potential ability of the fungus to cause disease on young and actively growing apple trees, it will be a serious threat to apple cultivation and industry.

## Introduction

The domesticated apple (*Malus* × *domestica* Borkh.) is one of the oldest, most popular, and widely grown temperate fruit crops in the world^1,2^. It is one of the most economically important fruit crops and ranks as the 3rd most produced fruit crop worldwide^3^. The fruits are predominantly used for the fresh market, even though other uses are cider production and processing^4–6^. Apple is an ancient fruit crop in Iran, growing in different locations from the northern to the western and central parts of the country^7,8^. A high level of genetic diversity is seen in cultivated apples in Iran, and the results of a phylogenetic study showed that Iran could be a paramount center of diversity for domesticated apples and an important center for domestication and passing on from Central Asia to the West via the Silk Routes^8,9^.

In Iran, West Azarbaijan province is the main apple-growing region with 63,661 ha and a total production of 1,118,285 metric tons in 2020, ranked first with 26.5% of the total production^10^. Apple trees have a long juvenile phase and often start bearing fruits after five years. For this reason, growers typically plant and grow a small number of well-assessed and historically successful apple varieties^2^. Two apple cultivars, ‘Red Delicious’ and ‘Golden Delicious’, are the main commercially grown apples in this region making up about 90% of apple cultivation.

Apple trees are affected by different fungal diseases; among them, stem and trunk canker as well as dieback diseases are of great importance, causing progressive losses over the years^11–18^. Depending on the incidence and severity of the infection, the disease impacts range from decreased yield with poor fruit quality and plant longevity to complete loss of fruits and trees, resulting in significant economic losses to growers. It has been estimated that abiotic and biotic stresses reduce the annual apple harvest by 12–25%^19^.

*Cytospora* species are important plant pathogens associated with branch dieback and canker disease on a wide range of plants with worldwide distribution^20–23^. They are usually considered as wound pathogens, invading host tissues through cracks, wounds, or other openings in the bark, leading to growth weakness and death of plants^21,24–26^. The fungal hyphae invade host tissues, decompose the cambium, and penetrate extensively into the phloem and xylem of trunks, twigs, and scaffold limbs, leading to perennial and latent infections providing a potential source of inoculum^27–30^.

Thus far, about 29 species of *Cytospora* have been reported from *Malus* spp. worldwide^30–34^, of which 19 species have been identified in Iran^32,34–36^. However, the taxonomic status of most of these species has not been confirmed through molecular approaches. Incorrect diagnosis and treatment of plant diseases generally result in the rapid spread of the diseases, and minor instances of these diseases can quickly become significant and costly problems under the pathogens’ rapid multiplication and conducive environmental conditions^37^. Due to the overlapping morphological characteristics, poor condition of single-gene phylogeny, and insufficiency of freshly collected specimens, multiphase approaches and multi-gene phylogeny have been suggested to elucidate accurate species boundaries among *Cytospora* isolates^21,26,38^.

During the past two decades and mainly due to climate change, apple orchards in West Azarbaijan province have been under severe threat from both biotic and abiotic agents^18,34,39^. Diplodia canker, die-back, decline, and root rots caused by different fungal pathogens, apple scab, and powdery mildew are the most prevalent fungal diseases of apple trees in the province^15,18,39,40^. In the course of our investigations on apple diseases in West Azarbaijan province, Iran, we observed disease symptoms including crown and collar canker and necrosis in three young apple orchards in Urmia, leading to relatively rapid tree decline and death. A fungus with *Cytospora* characteristics was frequently isolated from the diseased samples. The objective of this study was to (1) identify *Cytospora* species involved in the disease based on morphological characteristics and molecular multi-gene phylogeny, (2) assess the pathogenicity of the isolates on apple cv. ‘Red Delicious’ and (3) a preliminary evaluation of the reaction of 12 different apple cultivars to five selected isolates of the pathogen with higher aggressiveness.

## Results

### Disease symptoms, incidence, and fungal isolations

**Figure 1:**
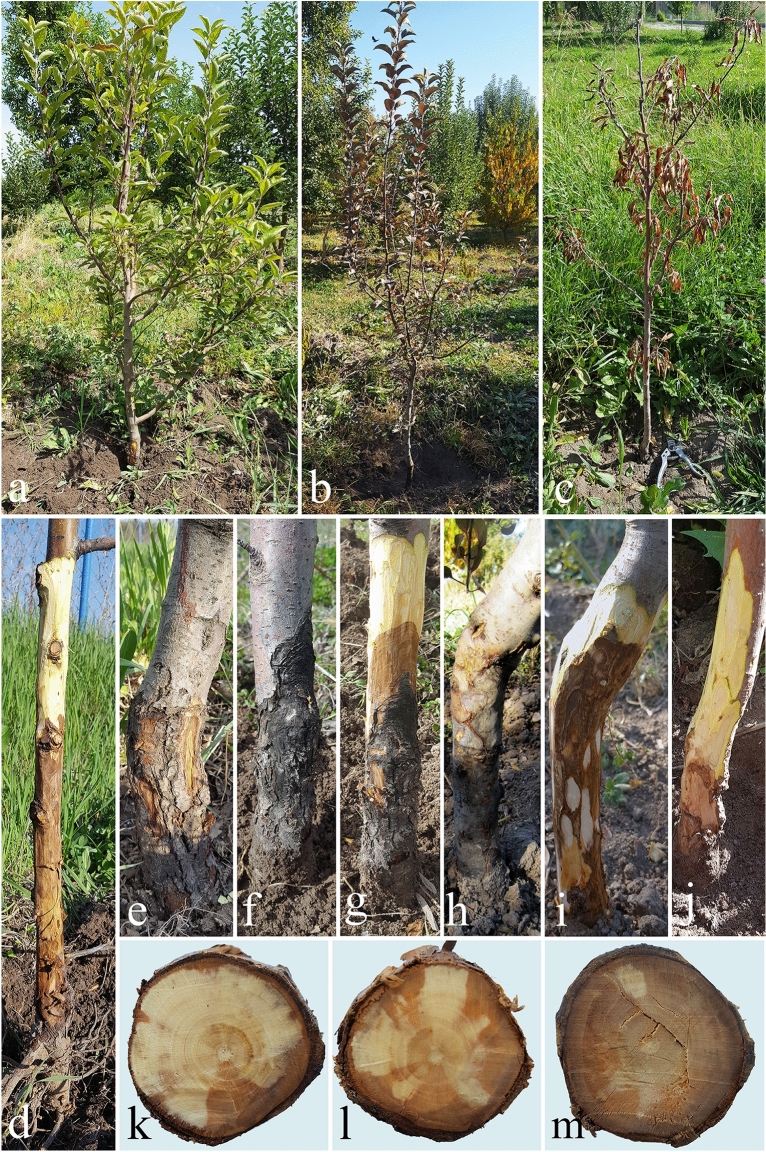
Typical symptoms of crown and collar canker and necrosis on naturally infected young apple trees cvs. ‘Red Delicious’ and ‘Golden Delicious’. (**a**, **b**) cv. ‘Red Delicious’. (**c**) cv. ‘Golden Delicious’. (**d**–**i**) Disease symptoms on the crown, collar and trunk of the cvs. ‘Red Delicious’ and (**j**) ‘Golden Delicious’. (**k**–**m**) Cross sections showing disease progress in the infected trunks of the cv. ‘Red Delicious’.

Characteristic external disease symptoms including general decline, cankers, and plant death were observed during the summer and early autumn in three young apple orchards, both on the cvs. ‘Golden Delicious’ and ‘Red Delicious’. The leaves in some individual branches were pale yellow in the beginning, then their margins became necrotic, and in late summer and early autumn, the color of the leaves turned purple and finally died (Fig. 1). Shoot elongation was arrested in affected plants. The bark of the diseased plants was discolored and sunken at the soil line, longitudinal cracks and cankers were formed on the bark surface and discoloration was extended progressively both upward (up to 50 cm from the graft union) and downward to the main roots and into the wood. A distinct margin separated the healthy bark tissue from the infected one and trees were killed when the infected area girdled the entire trunk base. In cross-sections, there was a light brown to brown discoloration and necrosis as V or U shape in the hardwood (Fig. 1). Based on these external symptoms in the surveyed orchards, the incidence of the disease on the cv. ʻRed Delicious’ (15%) was higher than the cv. ‘Golden Delicious’ (1%).

In this study, 24 fungal isolates (19 from the cv. ʻRed Delicious’ and five from the cv. ʻGolden Delicious’) were obtained and purified (Table 1). Based on the comparison of morphological characteristics and banding patterns generated from the ISSR-PCR of the purified isolates, three isolates were selected for multi-gene phylogenetic analyses and accurate species identification.

**Table 1:** Source, location and mean lesion lengths (mm) of 24 isolates of *Cytospora balanejica* on detached shoots of apple cv.

Isolate	Source/location	Mean lesion length (mm)
BA 2-4	Red Delicious, Balanej village (Orchard 1)	172^a^
KU 1-1	Golden Delicious, Kurane Village	148^b^
BA 2-1	Red Delicious, Balanej village (Orchard 1)	102^c^
BA 3-1	Red Delicious, Balanej village (Orchard 2)	92^d^
BA 1-1	Red Delicious, Balanej village (Orchard 2)	82^e^
KU 1-2	Red Delicious, Kurane village	75^f^
BA 3-2	Golden Delicious, Balanej village (Orchard 1)	75^f^
KU 2-3	Golden Delicious, Kurane Village	75^f^
BA 2-3	Red Delicious, Balanej village (Orchard 2)	74^g^
BA 2-2	Red Delicious, Balanej village (Orchard 1)	73^h^
BA 3-3	Red Delicious, Balanej village (Orchard 2)	71^i^
KU 2-1	Red Delicious, Kurane Village	70^j^
KU 1-3	Red Delicious, Kurane Village	68^k^
BA 5-1	Red Delicious, Balanej village (Orchard 1)	66^l^
KU 2-2	Red Delicious, Balanej village (Orchard 2)	65^m^
BA 1-2	Red Delicious, Balanej village (Orchard 1)	65^m^
BA 5-2	Golden Delicious, Balanej village (Orchard 1)	62^n^
BA 5-3	Red Delicious, Balanej Village (Orchard 1)	58^o^
KU 3-2	Red Delicious, Kurane Village	57^p^
KU 3-1	Red Delicious, Kurane Village	55^q^
BA 1-3	Red Delicious, Balanej village (Orchard 1)	52^r^
BA 4-1	Red Delicious, Balanej village (Orchard 2)	50^s^
BA 4-3	Red Delicious, Balanej village (Orchard 1)	46^t^
BA 4-2	Golden Delicious, Balanej village (Orchard 2)	45^u^

‘Red Delicious’ 21 days post-inoculation based on Duncan’s multiple range test. Different letters show significant differences at *P* ≤ 0.05.

### Phylogenetic analyses

**Figure 2:**
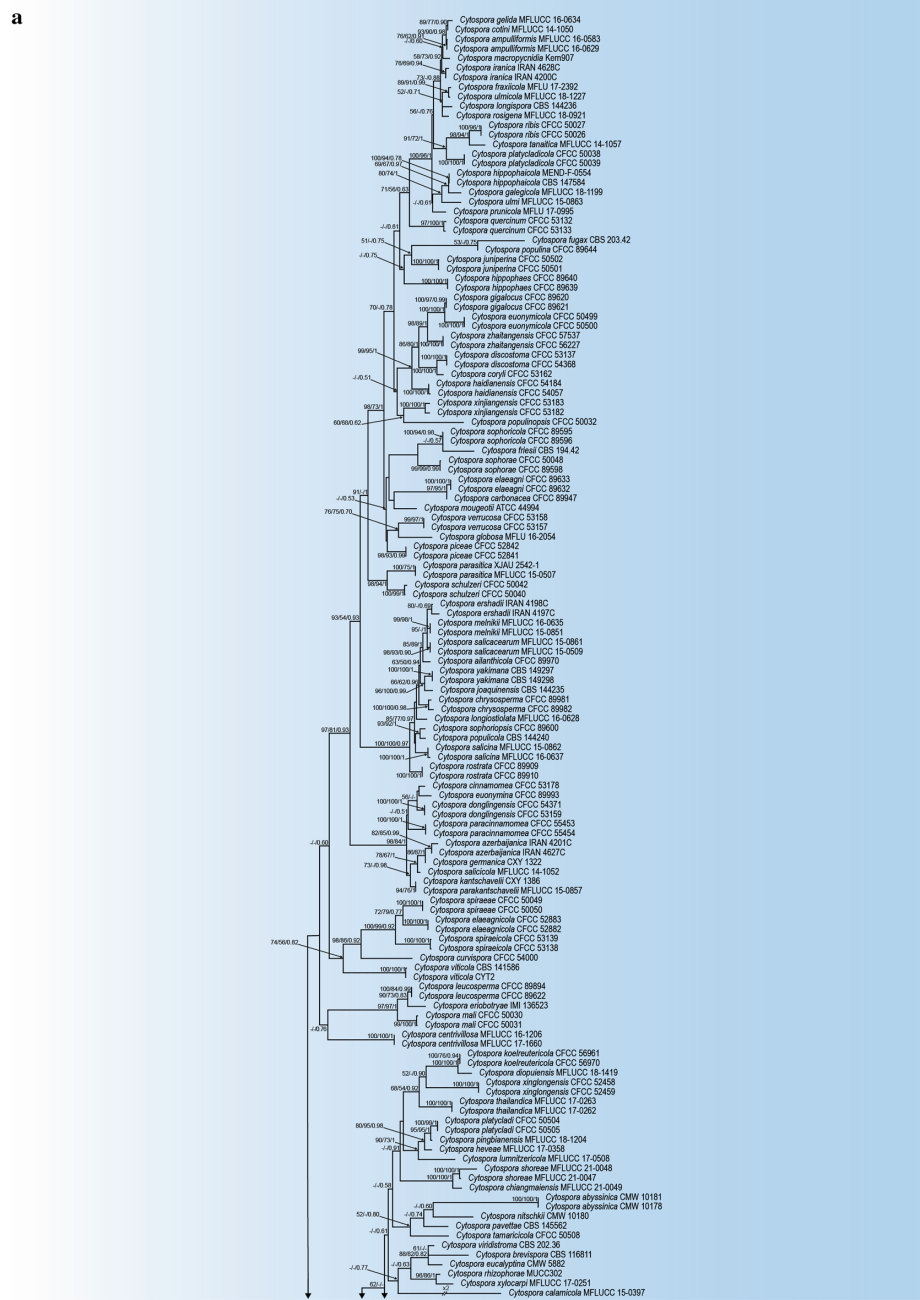

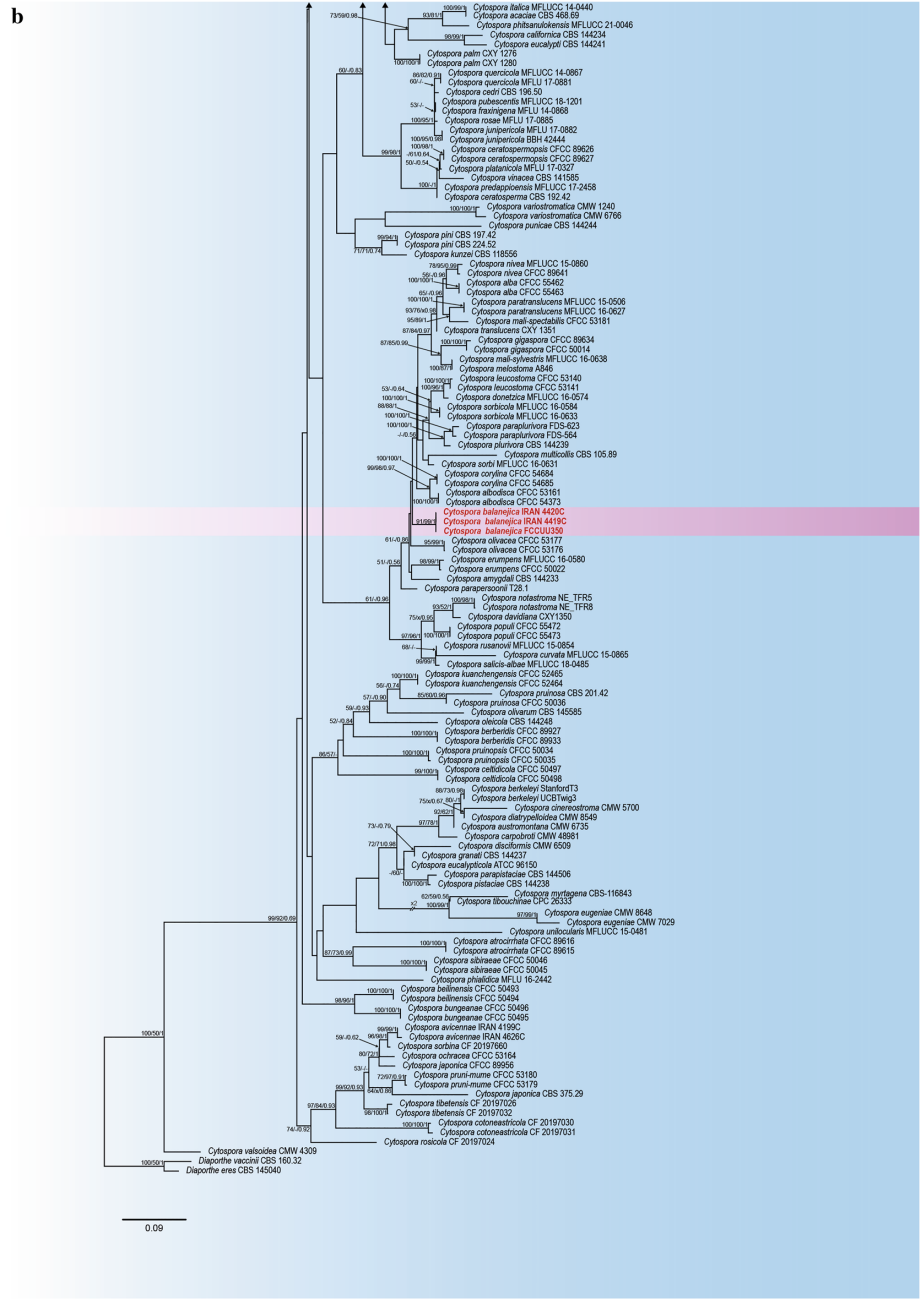
Maximum Likelihood (ML) tree based on combined ITS, LSU, *rpb2*, *act1* and *tef 1-a* sequences matrix in different *Cytospora* species. The Maximum Likelihood, Maximum Parsimony (MP) bootstrap support values and posterior probabilities of Bayesian inference (BIPP) > 50% are given at the nodes (ML/MP/BI). The tree was rooted to *Diaporthe eres* CBS 145040 and *Diaporthe vaccinii* CBS 160.32. The scale bar indicates the number of nucleotide substitutions.

The phylogenetic analyses of the combined dataset (ITS, LSU, *act1*, *rpb2*, and *tef 1-a*) include 257 *Cytospora* ingroup strains representing 175 *Cytospora* species and *Diaporthe eres* CBS 145040 and *Diaporthe vaccinii* CBS 160.32 as outgroup strains with a total of 3049 characters (1641 constant sites, 1408 variable sites, 240 parsimony-uninformative sites and 1168 parsimony-informative sites) including gaps (642 for ITS, 574 for LSU, 354 for *act1*, 743 for *rpb2*, and 763 for *tef1-α*) (Table 2). The results of best-fit substitution model evaluation in MrModeltest v2.3 recommended GTR+I+G, GTR+I+G, GTR+I+G, GTR+I+G and HKY+I+G models for ITS, LSU, *act1*, *rpb2* and *tef 1-a*, respectively (Table 2). The best-scoring RaxML tree with the final ML optimization likelihood value of − 47725.166937 (ln) is selected to denote and consider the phylogenetic relationships among the strains (Table 2, Fig. 2). The estimated base frequencies were as follows: A = 0.242358, C = 0.268654, G = 0.259113, T = 0.229874; substitution rates AC = 1.549133, AG = 3.919749, AT = 1.913775, CG = 1.087338, CT = 8.678832, GT = 1.000000; gamma distribution shape parameter α = 0.793946. A summary of phylogenetic information and substitution models for each dataset is provided in Table 2. Topologies of the individual gene trees were determined to be congruent and no conflicts were observed in species delimitation (data not shown). The phylogenetic trees generated from MP (TL = 9556; CI = 0.262; RI = 0.766; HI = 0.738) and BI analyses were topologically similar to the one generated via the ML analysis, and the latter is shown in Fig. 2. *Cytospora balanejica* represented a monophyletic clade with a high support value (ML/MP/BI = 91/99/1) (marked in pink in Fig. 2).

**Table 2:** Phylogenetic information of individual and combined sequence datasets used in phylogenetic analyses.

Parameter	ITS-rDNA	LSU	*act1*	*rpb2*	*tef 1-a*	Combined
Number of Taxa	256	175	183	159	157	257
Total characters	642	547	354	743	763	3049
Constant sites	360	420	150	435	276	1641
Variable sites	282	127	204	308	487	1408
Parsimony informative sites	217	59	183	272	437	1168
Parsimony uninformative sites	65	68	21	36	50	240
AIC Substitution Model^a^	GTR+I+G	GTR+I+G	GTR+I+G	GTR+I+G	HKY+I+G	GTR+I+G
Lset nst, Rates	6, invgamma	6, invgamma	6, invgamma	6, invgamma	2, invgamma	6, invgamma
-lnL	7224.308797	2313.705431	6383.267168	10,954.098764	17,674.526253	47,725.166937

^a^Akaike Information Criterion Substitution models implemented in Bayesian Inference.

### Taxonomy

**Figure 3:**
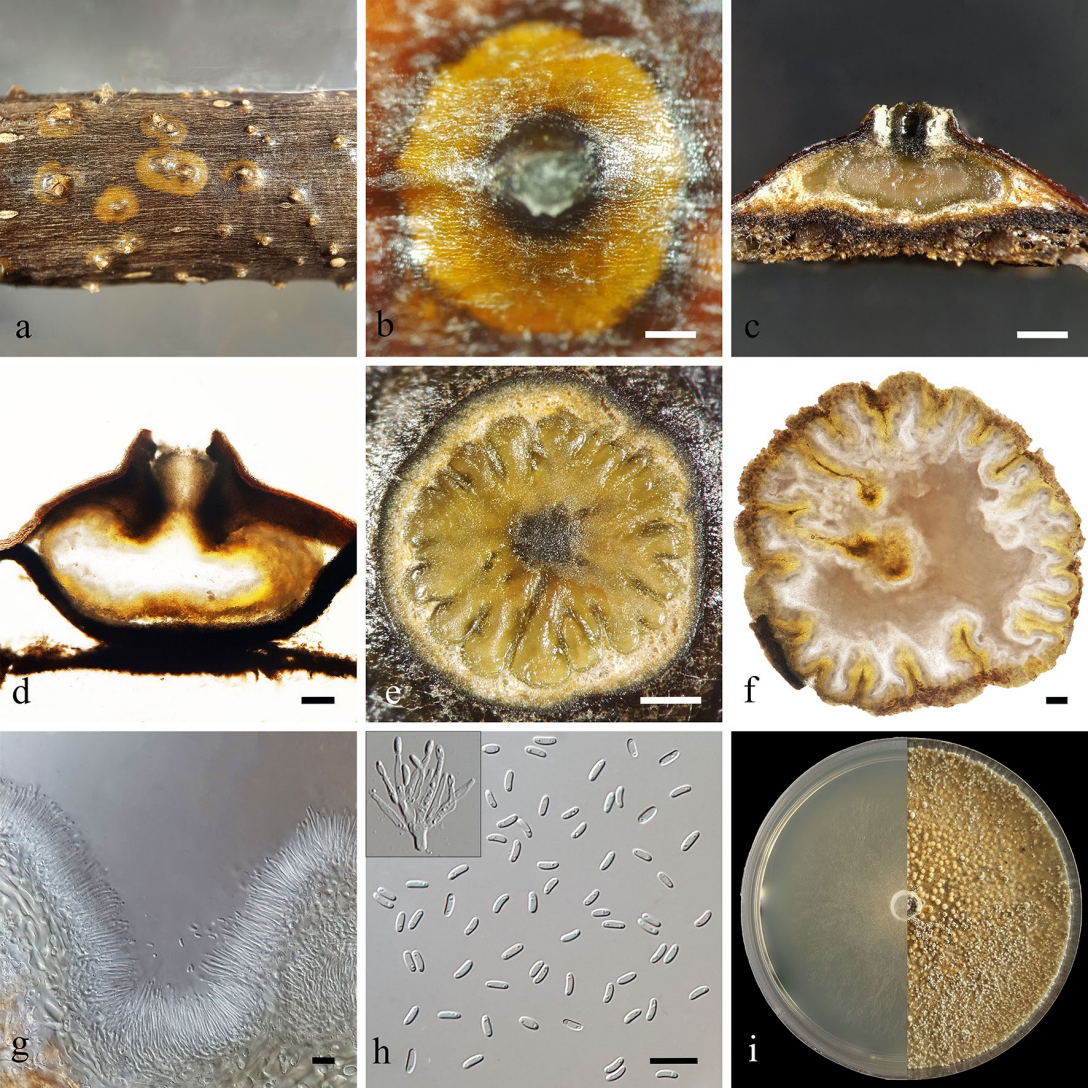
Morphology of *Cytospora balanejica* (IRAN 4419C). (**a**, **b**) Habit of conidiomata on twig. (**c**, **d**) Longitudinal section through conidiomata. (**e**, **f**) Transverse section of conidiomata. (**g**) Conidiophores and conidiogenous cells. (**h**) Conidia. (**i**) Colonies on PDA at 3 days (left) and 30 days (right). Scale bars: (**b**, **c**) and € = 250 µm; (**f**, **d**) = 100 µm and (**g**, **h**) = 10 µm.

*Cytospora balanejica* R. Azizi, Y. Ghosta & A. Ahmadpour sp. nov. (Fig. 3).

*MycoBank No.*: MB843116.

*Etymology*: Named after the locality, Balanej village, where the holotype was collected.

*Typification*: Iran, West Azarbaijan Province, Urmia City, Balanej Village, 37°23′50.4″ N, 45°09′15.9″ E., from crown of *Malus* × *domestica* cv. ʻRed Delicious’, 15 Oct. 2017, R. Azizi (Holotype: IRAN 18133F; ex-type living culture: IRAN 4419C).

*Description*: Asexual morph: Conidiomata labyrinthine cytosporoid, immersed in the bark, erumpent when mature through the surface of the bark, discoid to conical, pale luteous to luteous, with multiple locules, (800–)850–1490(–1700) µm in diam. Conceptacle conspicuous, black, circular, surrounded the stromata. Ectostromatic disk greenish black to black, circular to ovoid, (473–)563–802(–845) µm in diam., with a single ostiole per disk in the center. Ostiole conspicuous, circular to ovoid, olivaceous grey, at the same level as the disk surface, (94–)101–215(–230) µm in diam. Locules numerous, arranged circularly with shared invaginated walls. Conidiophores borne along the locules, hyaline, smooth, thin-walled, unbranched, or occasionally branched at the base. Conidiogenous cells entroblastic, phialidic, subcylindrical to cylindrical, tapering towards apices, (6.2–)9–17(–19) × (1–)1.2–2 µm. Conidia hyaline, smooth, elongate allantoid, mostly biguttulate, aseptate, 3–5 × 1–1.8 µm. Sexual morph: not observed.

*Culture characteristics*: Colonies after 3 days at 25 °C on PDA average 57 mm and entirely covering the 90-mm diam. Petri dish after 7 days, margin entire, white to buff, with scattered aerial hyphae at the center, the hyphae becoming very dense, pale luteous at center and honey at margins, forming abundant solitary or rarely aggregated pycnidia surrounded by off-white hyphae with age. Hyphae hyaline to light brown, septate, smooth-walled, and branched.

*Habitat and distribution*: Known only on *Malus* × *domestica* in Urmia, Iran.

*Additional specimens examined*: Iran, West Azarbaijan Province, Urmia City, Balanej Village, 37°24′26.1″ N, 45°10′24.8″ E., from the trunk of *Malus* × *domestica* cv. ‘Red Delicious’, 15 Oct. 2017, R. Azizi, (IRAN 4420C); West Azarbaijan Province, Urmia City, Kurane Village, 37°24′44.4″ N 45°8′45.3′′ E., from the trunk of *Malus* × *domestica* cv. ‘Golden Delicious’, 12 Sept. 2018, R. Azizi, (FCCUU 350).

*Notes*: *Cytospora balanejica* was isolated from young, declining apple trees showing symptoms of crown and collar canker and necrosis. The phylogenetic inferences based on the combined multi-gene phylogeny resolved this species as a monophyletic lineage distinct from all other strains included in this study, but closely related to a clade containing *C. albodisca* M. Pan & X.L. Fan and *C. corylina* H. Gao & X.L. Fan (Fig. 2). However, *C. balanejica* differs from *C. albodisca* based on the absence of ascomata and smaller conidia (3–5 × 1–1.8 µm vs. 5–7 × 1–2 µm in *C. albodisca*)^26^. Also, it differs from *C. corylina* based on the formation of distinct conceptacle, larger conidiomata (800–1700 µm vs. 850–1280 µm) and shorter conidia (3–5 µm vs. 3.5–7.5 µm in *C. corylina*)^41^. Pairwise sequence comparisons of the genomic regions in *C. balanejica* strain IRAN 4419C showed considerable nucleotide differences from *C. albodisca* strain CFCC 53161 (including 2 out of 466 in ITS, 30 out of 726 in *rpb2*, 20 out of 160 in *act1* and 73 out of 516 in *tef1-α*) and *C. corylina* strain CFCC 54684 (including 2 out of 466 in ITS, 30 out of 726 in *rpb2*, 17 out of 161 in *act1* and 75 out of 513 in *tef1-α*). Therefore, we describe *C. balanejica* here as a new species.

### Pathogenicity trials

**Figure 4:**
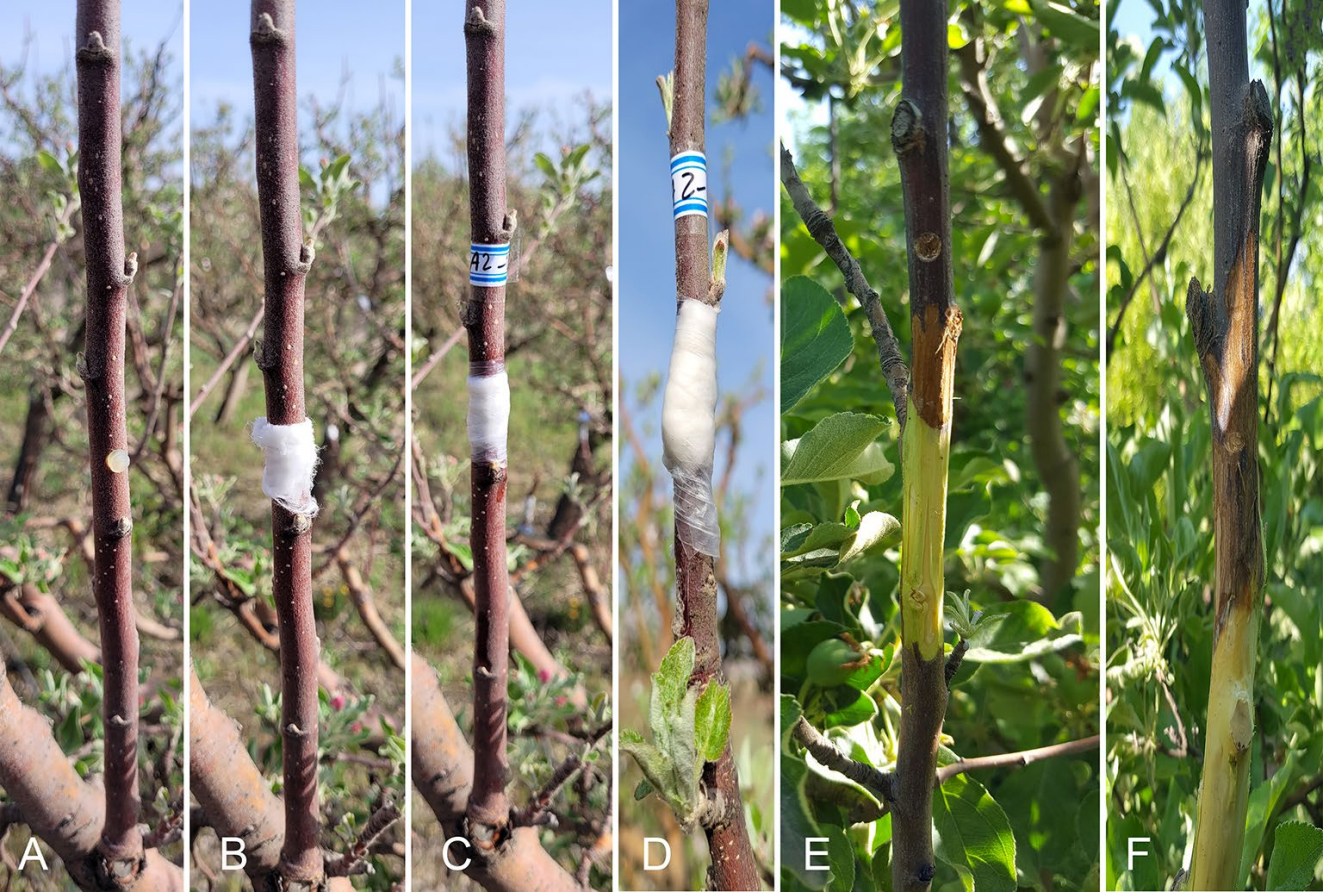
Pathogenicity tests of the most virulent isolate (BA 2-4) on apple cv. ‘Red Delicious’ under field conditions. (**A**–**D**) Inoculation process. (**E**, **F**) Bark and wood discoloration and necrosis 45 days post inoculation.

**Figure 5:**
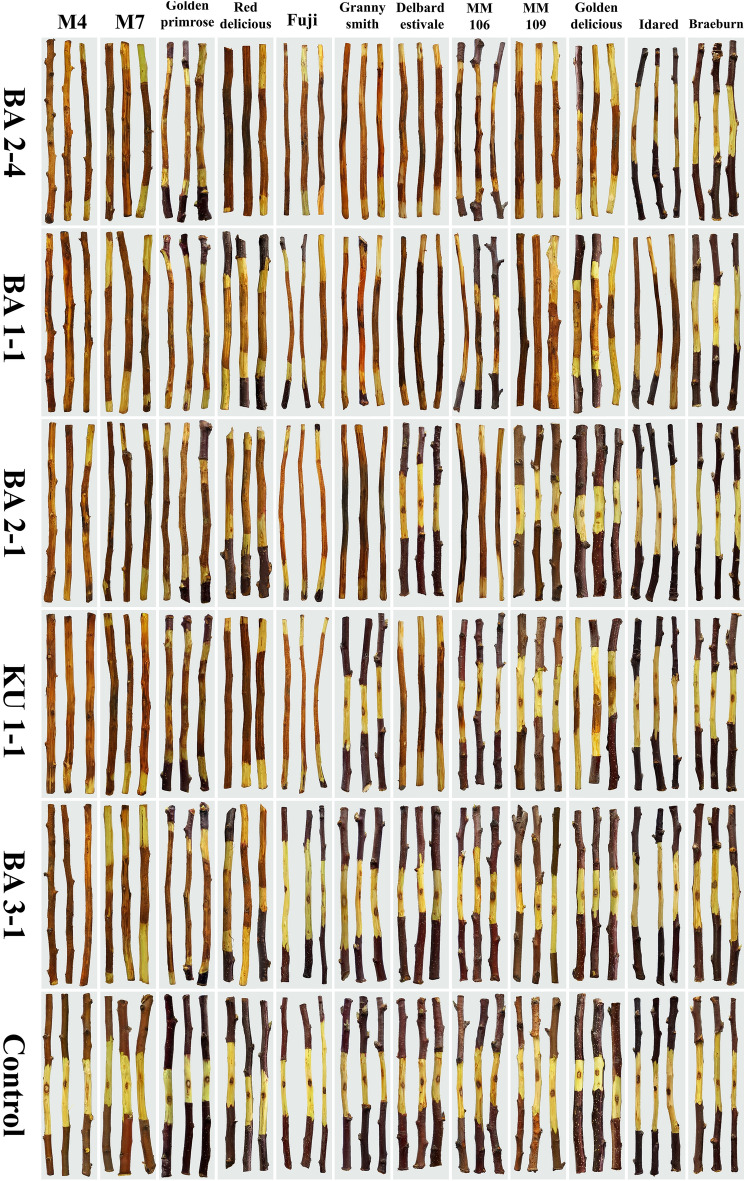
Pathogenicity tests of five selected isolates (BA 1-1, BA 1-2, BA 2-4, BA 3-1, and KU 1-1) of *Cytospra balanejica* against 12 apple cultivars.

**Figure 6:**
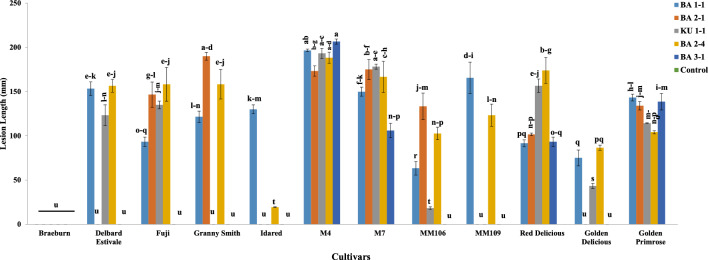
Mean lesion lengths (mm) of 12 apple cultivars inoculated with five selected isolates of *Cytospora balanejica* based on Duncan’s multiple range test. Different letters show statistically significant differences at *P* ≤ 0.05.

Results of pathogenicity tests of the isolates (24 isolates) on shoots of the cv. ‘Red Delicious’ showed sunken discolored lesions around the inoculated sites 14 days post-inoculation. Bark and wood discoloration was extended progressively upward and downward the inoculation site and after 20 days, fungal pycnidia were formed on the discolored bark. Despite this, the mean length of necrotic lesions varied among the isolates and ranged from 45 to 172 mm (Table 1). Also, the results of pathogenicity tests of the most virulent isolate (BA 2-4) under field conditions clearly showed bark and wood discoloration and necrosis 45 days post-inoculation (Fig. 4). Re-isolation of the inoculated fungus and re-identification based on morphological characteristics fulfilled Koch’s postulates. All negative controls were asymptomatic and no colonies were obtained from samples taken from the controls. The reaction of 12 tested cultivars against five selected isolates with the highest virulence showed that the interaction between the factors isolates × cultivars was varied and significantly different at *P* ≤ 0.05 (Figs. 5 and 6). Except for the cv. ‘Braeburn’ which did not produce any symptoms of infection similar to control treatment against all tested fungal isolates, the other cultivars showed symptoms of infection at least against two fungal isolates (Fig. 5). The mean length of necrotic lesion ranged from 19.3 mm (the cv. ‘Idared’) to 188.3 mm (the cv. ‘M4’) for isolate BA 2-4 and from 63.3 mm (the cv. ‘MM106’) to 196.6 mm (the cv. ‘M4’) for isolate BA 1-1, both isolates were obtained from the cv. ‘Red Delicious’ (Fig. 6). The mean length of necrotic lesions ranged from 18.3 mm (the cv. ‘MM106’) to 193.3 mm (the cv. ‘M4’) for isolate KU 1-1 which was isolated from the cv. ‘Golden Delicious’, although the cvs. ‘Granny Smith’, ‘MM109’, and ‘Idared’ did not show any symptoms of infection against this isolate (Fig. 6). Also, the cvs. ‘Delbard Estivale’, ‘MM109’, ‘Idared’, and ‘Golden Delicious’ did not develop any symptoms of infection against BA 2-1 isolate and the mean length of necrotic lesion ranged from 101.6 mm (the cv. ‘Red Delicious’) to 190 mm (the cv. ‘Granny Smith’). At last, only four cultivars including ‘M4’, ‘M7’, ‘Golden Primrose’, and ‘Red Delicious’ developed symptoms of infection against BA 3-1 isolate and the mean length of necrotic lesion ranged from 93.3 mm (the cv. ‘Red Delicious’) to 206.6 mm (the cv. ‘M4’) (Fig. 6). Moreover, the aggressiveness of five tested isolates was varied and the isolates BA 2-4 and BA 3-1 had the highest and lowest aggressiveness against 12 tested cultivars, respectively.

## Discussion

In this study, we found a new species of *Cytospora*, *C. balanejica*, associated with crown and collar canker and decline symptoms in young apple trees. The incidence of the disease was greater on the cv. ‘Red Delicious’ than the cv. ‘Golden Delicious’, indicating higher susceptibility of the first cultivar to this new *Cytospora* species. Our pathogenicity tests confirmed this, as all studied isolates were pathogenic on the shoots of the cv. ‘Red Delicious’ and had greater virulence (longer necrotic lesions) than the cv. ‘Golden Delicious’ (Fig. 6). Although the disease incidence was significantly lower in the cv. ‘Golden Delicious’ than the cv. ‘Red Delicious’, it is important to note that the infected plants can provide an inoculum reservoir for the pathogen.

The results of our study showed that the cv. ‘Braeburn’ did not develop any symptoms of infection against all the tested fungal isolates, suggesting that it might have some levels of resistance to the disease. The other 11 examined cultivars showed lesions with various degrees of severity and could be considered susceptible. The resistance of 53 accessions of diverse *Malus* species and their interspecific hybrids was tested against *Valsa ceratosperma* (syn.: *Cytospora ceratosperma*) using excised shoot assay and by measuring the length of necrotic lesion^42^. Fourteen accessions were evaluated as resistant and the highest level of resistance was identified in *Malus sieboldii* Rehder, which was effective against different isolates of the tested fungus. Similar results were also found in pathogenicity studies using different fungal species and host plants^43–48^. The virulence of the tested fungal isolates as measured by lesion length was varied and this could be attributed to the genetic diversity among the isolates. Variability in the lesion length has been reported in pathogenicity evaluations of the isolates of *Cytospora* spp. and other fungal pathogens^31,46,49–53^.

*Cytospora* species generally cause canker, dieback and decline diseases with different symptoms on a wide range of woody perennials including fruit and nut trees, forest and urban trees, and rarely on herbaceous plants with strong ecological adaptability^21,26,41,54,55^. In our study, disease symptoms differed from the previously reported symptoms of apple canker diseases caused by *Cytospora* species, as the disease starts from the crown and collar region of apple trees (Fig. 1). Other apple diseases such as Neonectria canker, Phytophthora crown, collar and root rots, Rosellinia root rot and fire blight have been reported in the literature to cause similar symptoms on affected young apple trees^13,56^. The similarity in symptoms caused by *C. balanejica* and other diseases, especially in the early stages of disease development, makes it difficult to accurately identify the causal agents without further laboratory examination.

Apple is one of the main hosts that suffer severe damage from the Cytospora canker disease^31,57,58^. In a most recent study, eight species of *Cytospora* were identified from apple trees in Iran^34^, emphasizing the necessity of extensive pathogen surveys in apple production regions. Understanding the exact diversity of pathogenic fungi such as *Cytospora* spp. is crucial for devising regional management strategies for each species, developing rapid diagnostic tools, screening for resistance, and accomplishing regulatory control measurements^59^. Earlier species identification in *Cytospora* has relied on morphological characteristics and host associations; however, these characteristics are not stable and informative, confusing species identification and delimitation^41,55,60^.

The results of our phylogenetic analyses using ITS-rDNA sequences placed *C. balanejica* together with *C. albodisca*, *C. corylina*, and *C. olivacea* in an unresolved clade, confirmed the poor utility of this genomic region in the differentiation of *Cytospora* species^21,38,61,62^ (Supplementary Fig. S1). Recent studies using a polyphasic approach, morphology, and multi-gene phylogeny, have revealed hidden fungal diversity, and led to the description of several new cryptic *Cytospora* species^21,23,26,38,41^. Based on our multi-gene phylogenetic analyses, *C. balanejica* formed a well-defined monophyletic lineage distinct from all other strains with close affinity to *C. albodisca* and *C. corylina*, two recently described *Cytospora* species (Fig. 2). *Cytospora albodisca* and *C. corylina* were isolated from the branches of *Platycladus orientalis* (L.) Franco and *Corylus heterophylla* Fisch. ex. Trautv. in China showing canker and dieback symptoms, respectively^26,41^.

This study found that apple trees are hosts of a new pathogenic species of *Cytospora*, which should be considered a potentially important causal agent of apple crown and collar canker disease in the studied area. Because *Cytospora* species are generally considered wound pathogens, infecting plants through cracks and wounds caused by freezing injuries, leaf scars, sunburn, oil injuries, shade-weakened twigs, and pruning wounds^21,53,63^, more precautions should be taken during grafting. Even though this study extends our knowledge about the role of a new *Cytospora* species in crown and collar canker disease on young apple trees, more studies are needed to reveal its biology and ecology, assess the susceptibility/resistance of apple cultivars under field conditions, and its host range and epidemiology to the development of effective management strategies.

## Material and methods

### Collection of samples and fungi isolation

Young apple trees (2–6 years old) showing symptoms of decreased growth, decline, and death from three orchards in Urmia, West Azarbaijan province, Iran, were evaluated. Samples were collected from the crown, collar, and trunk base showing bark and wood discoloration and canker (Fig. 1), placed separately in clean paper bags, and transferred to the laboratory for further investigation. Samples were washed gently under running tap water, then cut into smaller pieces (1 × 1 cm^2^) from the interfaces of the healthy and diseased tissues, and surface disinfested in 3% sodium hypochlorite solution for 2 min, rinsed again three times with sterile distilled water and blotted dry on autoclave sterilized filter paper. The pieces were plated onto potato-dextrose-agar medium supplemented with streptomycin sulfate and penicillin G (150 ppm each) to inhibit bacterial growth (PDA; Merck, Darmstadt, Germany) in 90 mm diam. glass Petri dishes. Petri dishes were incubated at 25 ± 1 °C in darkness, examined at 24 h intervals and hyphae growing out from the plant tissues were transferred to fresh PDA. Pure cultures were obtained using the hyphal tip method. The purified isolates were maintained on PDA slants containing a piece of filter paper and stored at 4 °C. The isolates were deposited in the Fungal Culture Collection of the Iranian Research Institute of Plant Protection (“IRAN”) and the Fungal Culture Collection of Urmia University (FCCUU).

### Plant materials

It is noted that plant materials used in this study were legitimate samples from apple orchards and all methods comprising plant studies were performed following the relevant guidelines, regulations, and legislation. Required permission to collect samples of apple trees from various orchards in Urmia, West Azarbaijan province, was obtained.

### DNA extraction, PCR amplification, sequencing, and phylogenetic analysis

Total genomic DNA was extracted from the mycelial mass of fungal isolates cultured in potato dextrose broth (PDB, Mirmedia Microbiology, Iran) for 7–10 days using the Exgene™ Cell SV mini kit (GeneAll Biotechnology Co, South Korea) following the manufacturer’s instruction. For the preliminary screening of all recovered isolates, polymorphic banding patterns of the isolates were generated by an ISSR-PCR method with ISSR5 ((GA)_5_YC) primer and were compared. Each polymerase chain reaction (PCR) mixture contained 0.4 μM of the primer, 4 μL of a ready master mix (Taq DNA polymerase 2× Master Mix Red, 2 mM MgCl_2_, Ampliqon Company, Denmark), and about 10 ng of template DNA in a final volume of 10 μL. The thermal cycling condition consisted of an initial denaturation step of 5 min at 95 °C followed by 35 cycles of 45 s at 95 °C, 60 s at 41 °C and 90 s at 72 °C and a final extension step of 10 min at 72 °C. Amplicons were visualized on a 1% agarose gel. Isolates with the same banding pattern were considered as the same taxon. To reveal the phylogenetic relationship among the isolates, three isolates were selected based on ISSR banding pattern and morphological characteristics for multi-gene sequencing (Table 3). The internal transcribed spacer region of nuclear ribosomal DNA (ITS1-5.8S-ITS2), parts of the nuclear ribosomal large subunit (LSU), translation elongation factor 1-α (*tef1-α*), RNA polymerase II (*rpb2*) and actin (*act1*) genes were amplified using the primer pairs ITS5/ITS4^64^, LR0R/LR7^65^, EF1-728F/EF-2^66,67^, RPB2-5F2/fRPB2-7cR^68,69^ and ACT512F/ACT783R^67^, respectively. All primers were purchased from Pishgam Company, Tehran, Iran. The PCR mixtures for all reactions consisted of about 10 ng/µL of genomic DNA, 0.4 µM of each primer, and 12.5 µL of 2× ready-to-use reaction mix (Taq DNA polymerase 2× Master Mix Red, 2 mM MgCl_2_, Ampliqon, Denmark) in a total volume of 25 µL. Thermal conditions for PCR amplification of ITS, LSU, *act1*, and *tef1-α* consisted of an initial denaturation step of 5 min at 95 °C, followed by 35 cycles of 30 s at 95 °C, 30 s at 57 °C and 60 s at 72 °C, and a final extension step of 5 min at 72 °C. The part of the *rpb2* gene was amplified using touch-down PCR consisting of an initial denaturation step of 5 min at 95 °C, followed by 40 cycles of 45 s at 95 °C, 45 s at 60–55 °C (annealing temperature decreased 0.5 °C in the first 10 cycles), 45 s at 72 °C and a final extension step of 10 min at 72 °C. PCR products were visualized on a 1.5% Agarose gel (100 V for 30 min) stained with CyberSafe (Safe DNA Stain, 6X Pishgam, Iran), following the manufacturer’s instruction to confirm the amplicon presence and size. Amplification products were purified and sequenced by Macrogen Inc. (Seoul, South Korea).

**Table 3:** Fungal isolates used in the molecular analyses in this study and GenBank accession numbers.

Species	Strain^a^	Host	Origin	GenBank accession numbers				
				ITS	LSU	*act1*	*rpb2*	*tef1-α*
*Cytospora abyssinica*	CMW 10181^T^	*Eucalyptus globulus*	Ethiopia	AY347353	NA	NA	NA	NA
*C. abyssinica*	CMW 10178	*Eucalyptus globulus*	Ethiopia	AY347354	NA	NA	NA	NA
*C. acaciae*	CBS 468.69	*Ceratonia siliqua*	Spain	DQ243804	NA	NA	NA	NA
*C. ailanthicola*	CFCC 89970^T^	*Ailanthus altissima*	Ningxia, China	MH933618	MH933653	MH933526	MH933592	MH933494
*C. alba*	CFCC 55462^T^	*Salix matsudana*	Gansu, China	MZ702593	NA	OK303457	OK303516	OK303577
*C. alba*	CFCC 55463^T^	*Salix matsudana*	Gansu, China	MZ702594	NA	OK303458	OK303517	OK303578
*C. albodisca*	CFCC 53161^T^	*Platycladus orientalis*	Beijing, China	MW418406	MW418418	MW422899	MW422909	MW422921
*C. albodisca*	CFCC 54373	*Platycladus orientalis*	Beijing, China	MW418407	MW418419	MW422900	MW422910	MW422922
*C. ampulliformis*	MFLUCC 16-0583^T^	*Sorbus intermedia*	Russia	KY417726	KY417760	KY417692	KY417794	NA
*C. ampulliformis*	MFLUCC 16-0629	*Acer platanoides*	Russia	KY417727	KY417761	KY417693	KY417795	NA
*C. amygdali*	CBS 144233^T^	*Prunus dulcis*	California, USA	MG971853	NA	MG972002	NA	MG971659
*C. atrocirrhata*	CFCC 89615	*Juglans regia*	Qinghai, China	KR045618	KR045700	KF498673	KU710946	KP310858
*C. atrocirrhata*	CFCC 89616	*Juglans regia*	Qinghai, China	KR045619	KR045701	KF498674	KU710947	KP310859
*C. austromontana*	CMW 6735^T^	*Eucalyptus pauciflora*	Australia	AY347361	NA	NA	NA	NA
*C. avicennae*	IRAN 4199C^T^	*Malus domestica*	Nahavand, Iran	MW295650	NA	MZ014511	MW824358	MW394145
*C. avicennae*	IRAN 4626C	*Malus domestica*	Arak, Iran	OM368649	NA	NA	NA	OM372511
*C. azerbaijanica*	IRAN 4201C^T^	*Malus domestica*	Urmia, Iran	MW295526	NA	MZ014513	MW824360	MW394147
*C. azerbaijanica*	IRAN 4627C	*Malus domestica*	Miandoab, Iran	OM368650	NA	NA	NA	OM372512
***C. balanejica***	**IRAN 4419C**^**T**^	***Malus domestica***	**Urmia, Iran**	**MZ948960**	**MZ948957**	**MZ997842**	**MZ997845**	**MZ997848**
***C. balanejica***	**IRAN 4420C**	***Malus domestica***	**Urmia, Iran**	**MZ948961**	**MZ948958**	**MZ997843**	**MZ997846**	**MZ997849**
***C. balanejica***	**FCCUU 350**	***Malus domestica***	**Urmia, Iran**	**MZ948962**	**MZ948959**	**MZ997844**	**MZ997847**	**MZ997850**
*C. beilinensis*	CFCC 50493^T^	*Pinus armandii*	Beijing, China	MH933619	MH933654	MH933527	NA	MH933495
*C. beilinensis*	CFCC 50494	*Pinus armandii*	Beijing, China	MH933620	MH933655	MH933528	NA	MH933496
*C. berberidis*	CFCC 89927^T^	*Berberis dasystachya*	Qinghai, China	KR045620	KR045702	KU710990	KU710948	KU710913
*C. berberidis*	CFCC 89933	*Berberis dasystachya*	Qinghai, China	KR045621	KR045703	KU710991	KU710949	KU710914
*C. berkeleyi*	StanfordT3^T^	*Eucalyptus globulus*	USA	AY347350	NA	NA	NA	NA
*C. berkeleyi*	UCBTwig3	*Eucalyptus globulus*	USA	AY347349	NA	NA	NA	NA
*C. brevispora*	CBS 116811^T^	*Eucalyptus grandis tereticornis*	Congo	AF192315	NA	NA	NA	NA
*C. bungeana*	CFCC 50495^T^	*Pinus bungeana*	Shanxi, China	MH933621	MH933656	MH933529	MH933593	MH933497
*C. bungeana*	CFCC 50496	*Pinus bungeana*	Shanxi, China	MH933622	MH933657	MH933530	MH933594	MH933498
*C. calamicola*	MFLUCC 15-0397^T^	*Calamus* sp.	Phang-Nga, Thailand	ON650702	ON650679	NA	NA	NA
*C. californica*	CBS 144234^T^	*Juglans regia*	California, USA	MG971935	NA	MG972083	NA	MG971645
*C. carbonacea*	CFCC 89947	*Ulmus pumila*	Qinghai, China	KR045622	KP310812	KP310842	KU710950	KP310855
*C. carpobroti*	CMW 48981^T^	*Carpobrotus edulis*	South Africa	MH382812	MH411216	NA	NA	MH411212
*C. cedri*	CBS 196.50	NA	Italy	AF192311	NA	NA	NA	NA
*C. celtidicola*	CFCC 50497^T^	*Celtis sinensis*	Anhui, China	MH933623	MH933658	MH933531	MH933595	MH933499
*C. celtidicola*	CFCC 50498	*Celtis sinensis*	Anhui, China	MH933624	MH933659	MH933532	MH933596	MH933500
*C. centrivillosa*	MFLUCC 16-1206^T^	*Sorbus domestica*	Italy	MF190122	MF190068	NA	MF377600	NA
*C. centrivillosa*	MFLUCC 17-1660	*Sorbus domestica*	Italy	MF190123	MF190069	NA	MF377601	NA
*C. ceratosperma*	CBS 192.42	*Taxus baccata*	Switzerland	AY347333	NA	NA	NA	NA
*C. ceratospermopsis*	CFCC 89626^T^	*Juglans regia*	Shaanxi, China	KR045647	KR045726	KU711011	KU710978	KU710934
*C. ceratospermopsis*	CFCC 89627	*Juglans regia*	Shaanxi, China	KR045648	KR045727	KU711012	KU710979	KU710935
*C. chiangmaiensis*	MFLUCC 21-0049^T^	*Shorea* sp.	Chiang Mai,Thailand	MZ356514	MZ356518	MZ451157	MZ451165	MZ451161
*C. chrysosperma*	CFCC 89981	*Populus alba* subsp. *pyramidalis*	Gansu, China	MH933625	MH933660	MH933533	MH933597	MH933501
*C. chrysosperma*	CFCC 89982	*Ulmus pumila*	Tibet, China	KP281261	KP310805	KP310835	NA	KP310848
*C. cinereostroma*	CMW 5700^T^	*Eucalyptus globulus*	Chile	AY347377	NA	NA	NA	NA
*C. cinnamomea*	CFCC 53178^T^	*Prunus armeniaca*	Xinjiang, China	MK673054	MK673084	MK673024	NA	NA
*C. coryli*	CFCC 53162^T^	*Corylus mandshurica*	Beijing, China	MN854450	MN854661	NA	MN850751	MN850758
*C. corylina*	CFCC 54684^T^	*Corylus heterophylla*	Beijing, China	MW839861	NA	MW815937	MW815951	MW815886
*C. corylina*	CFCC 54685	*Corylus heterophylla*	Beijing, China	MW839862	NA	MW815938	MW815952	MW815887
*C. cotini*	MFLUCC 14-1050^T^	*Cotinus coggygria*	Russia	KX430142	KX430143	NA	KX430144	NA
*C. cotoneastricola*	CF 20197030	*Cotoneaster* sp.	Tibet, China	MK673074	MK673104	MK673044	MK673014	MK672960
*C. cotoneastricola*	CF 20197031^T^	*Cotoneaster* sp.	Tibet, China	MK673075	MK673105	MK673045	MK673015	MK672961
*C. curvata*	MFLUCC 15-0865^T^	*Salix alba*	Russia	KY417728	KY417762	KY417694	NA	NA
*C. curvispora*	CFCC 54000^T^	*Corylus heterophylla*	Beijing, China	MW839851	NA	MW815931	MW815945	MW815880
*C. davidiana*	CXY 1350^T^	*Populus davidiana*	Inner Mongolia, China	KM034870	NA	NA	NA	NA
*C. diatrypelloidea*	CMW 8549^T^	*Eucalyptus globulus*	Australia	AY347368	NA	NA	NA	NA
*C. diopuiensis*	MFLUCC 18-1419^T^	Undefined wood	Chiang Mai, Thailand	MK912137	MK571765	MN685819	NA	NA
*C. disciformis*	CMW 6509^T^	*Eucalyptus grandis*	Uruguay	AY347374	NA	NA	NA	NA
*C. discostoma*	CFCC 53137^T^	*Platycladus orientalis*	Beijing, China	MW418404	MW418416	MW422897	MW422907	MW422919
*C. discostoma*	CFCC 54368	*Platycladus orientalis*	Beijing, China	MW418405	MW418417	MW422898	MW422908	MW422920
*C. donetzica*	MFLUCC 16-0574^T^	*Crataegus monogyna*	Russia	KY417731	KY417765	KY417697	KY417799	NA
*C. donglingensis*	CFCC 53159^T^	*Platycladus orientalis*	Beijing, China	MW418412	MW418424	MW422903	MW422915	MW422927
*C. donglingensis*	CFCC 54371	*Platycladus orientalis*	Beijing, China	MW418413	MW418425	MW422904	MW422916	MW422928
*C. elaeagni*	CFCC 89632	*Elaeagnus angustifolia*	Ningxia, China	KR045626	KR045706	KU710995	KU710955	KU710918
*C. elaeagni*	CFCC 89633	*Elaeagnus angustifolia*	Ningxia, China	KF765677	KF765693	KU710996	KU710956	KU710919
*C. elaeagnicola*	CFCC 52882^T^	*Elaeagnus angustifolia*	China	MK732341	MK732338	MK732344	MK732347	NA
*C. elaeagnicola*	CFCC 52883	*Elaeagnus angustifolia*	China	MK732342	MK732339	MK732345	MK732348	NA
*C. eriobotryae*	IMI 136523^T^	*Eriobotrya japonica*	India	AY347327	NA	NA	NA	NA
*C. ershadii*	IRAN 4198C^T^	*Malus domestica*	Arak, Iran	MW295523	NA	MZ014510	MW824357	MW394144
*C. ershadii*	IRAN 4197C	*Malus domestica*	Nahavand, Iran	MW295510	NA	NA	NA	MW394143
*C. erumpens*	MFLUCC 16-0580^T^	*Salix* × *fragilis*	Russia	KY417733	KY417767	KY417699	KY417801	NA
*C. erumpens*	CFCC 50022	*Prunus padus*	Shanxi, China	MH933627	MH933661	MH933534	NA	MH933502
*C. eucalypti*	CBS 144241	*Eucalyptus globulus*	California, USA	MG971907	NA	MG972056	NA	MG971617
*C. eucalypticola*	ATCC 96150^T^	*Eucalyptus nitens*	Australia	AY347358	NA	NA	NA	NA
*C. eucalyptina*	CMW 5882	*Eucalyptus grandis*	Columbia	AY347375	NA	NA	NA	NA
*C. eugeniae*	CMW 7029	*Tibouchina* sp.	Australia	AY347364	NA	NA	NA	NA
*C. eugeniae*	CMW 8648	*Eugenia* sp.	Indonesia	AY347344	NA	NA	NA	NA
*C. euonymicola*	CFCC 50499^T^	*Euonymus kiautschovicus*	Shaanxi, China	MH933628	MH933662	MH933535	MH933598	MH933503
*C. euonymicola*	CFCC 50500	*Euonymus kiautschovicus*	Shaanxi, China	MH933629	MH933663	MH933536	MH933599	MH933504
*C. euonymina*	CFCC 89993^T^	*Euonymus kiautschovicus*	Shanxi, China	MH933630	MH933664	MH933537	MH933600	MH933505
*C. fraxiicola*	MFLU 17-2392	dead branches	Russia	NA	MN764356	MN995562	NA	NA
*C. fraxinigena*	MFLUCC 14-0868^T^	*Fraxinus ornus*	Italy	MF190133	MF190078	NA	NA	NA
*C. friesii*	CBS 194.42	*Abies alba*	Switzerland	AY347328	NA	NA	NA	NA
*C. fugax*	CBS 203.42	*Salix* sp.	Switzerland	AY347323	NA	NA	NA	NA
*C. galegicola*	MFLUCC 18-1199^T^	*Galega officinalis*	Forlì-Cesena, Italy	MK912128	MK571756	MN685810	MN685820	NA
*C. gelida*	MFLUCC 16-0634^T^	*Cotinus coggygria*	Russia	KY563245	KY563247	KY563241	KY563243	NA
*C. germanica*	CXY 1322	*Elaeagnus oxycarpa*	China	JQ086563	JX524617	NA	NA	NA
*C. gigalocus*	CFCC 89620^T^	*Juglans regia*	Qinghai, China	KR045628	KR045708	KU710997	KU710957	KU710920
*C. gigalocus*	CFCC 89621	*Juglans regia*	Qinghai, China	KR045629	KR045709	KU710998	KU710958	KU710921
*C. gigaspora*	CFCC 50014	*Juniperus procumbens*	Shanxi, China	KR045630	KR045710	KU710999	KU710959	KU710922
*C. gigaspora*	CFCC 89634^T^	*Salix psammophila*	Shaanxi, China	KF765671	KF765687	KU711000	KU710960	KU710923
*C. globosa*	MFLU 16-2054^T^	*Abies alba*	Italy	MT177935	MT177962	NA	MT432212	MT454016
*C. granati*	CBS 144237^T^	*Punica granatum*	California, USA	MG971799	NA	MG971949	NA	MG971514
*C. haidianensis*	CFCC 54057^T^	*Euonymus alatus*	China	MT360042	NA	MT363979	MT363988	MT363998
*C. haidianensis*	CFCC 54184	*Euonymus alatus*	Beijing, China	MT360043	NA	MT363980	MT363989	MT363999
*C. heveae*	MFLUCC 17-0358^T^	*Hevea brasiliensis*	Thailand	OL780505	OL782085	OL944407	NA	OL944428
*C. hippophaës*	CFCC 89639	*Hippophaë rhamnoides*	Gansu, China	KR045632	KR045712	KU711001	KU710961	KU710924
*C. hippophaës*	CFCC 89640	*Hippophaë rhamnoides*	Gansu, China	KF765682	KF765698	KF765730	KU710962	KP310865
*C. hippophaicola*	CBS 147584^T^	*Hippophae rhamnoides*	Czech Republic	MZ702814	MZ702873	MZ712150	MZ712160	MZ712155
*C. hippophaicola*	MEND-F-0554	*Vaccinium corymbosum*	Czech Republic	MZ702815	MZ702872	MZ712151	MZ712161	MZ712156
*C. iranica*	IRAN 4200C^T^	*Malus domestica*	Arak, Iran	MW295652	NA	MZ014512	MW824359	MW394146
*C. iranica*	IRAN 4628C	*Malus domestica*	Nahavand, Iran	OM368651	NA	NA	NA	OM372513
*C. italica*	MFLUCC 14-0440	*Tamarix gallica*	Italy	KU900329	KU900301	NA	NA	NA
*C. japonica*	CBS 375.29	*Prunus persica*	Japan	AF191185	NA	NA	NA	NA
*C. japonica*	CFCC 89956	*Prunus cerasifera*	Ningxia, China	KR045624	KR045704	KU710993	KU710953	KU710916
*C. joaquinensis*	CBS 144235^T^	*Populus deltoides*	California, USA	MG971895	NA	MG972044	NA	MG971605
*C. junipericola*	BBH 42444	*Juniperus communis*	Italy	MF190126	MF190071	NA	NA	MF377579
*C. junipericola*	MFLU 17-0882^T^	*Juniperus communis*	Italy	MF190125	MF190072	NA	NA	MF377580
*C. juniperina*	CFCC 50501^T^	*Juniperus przewalskii*	Sichuan, China	MH933632	MH933666	MH933539	MH933602	MH933507
*C. juniperina*	CFCC 50502	*Juniperus przewalskii*	Sichuan, China	MH933633	MH933667	MH933540	MH933603	MH933508
*C. kantschavelii*	CXY 1386	*Populus maximowiczii*	Chongqing, China	KM034867	NA	NA	NA	NA
*C. koelreutericola*	CFCC 56961^T^	*Koelreuteria paniculata*	Beijing, China	ON376918	NA	ON390905	ON390908	ON390914
*C. koelreutericola*	CFCC 56970	*Koelreuteria paniculata*	Beijing, China	ON376917	NA	ON390904	ON390907	ON390913
*C. kuanchengensis*	CFCC 52464^T^	*Castanea mollissima*	China	MK432616	MK429886	MK442940	MK578076	NA
*C. kuanchengensis*	CFCC 52465	*Castanea mollissima*	China	MK432617	MK429887	MK442941	MK578077	NA
*C. kunzei*	CBS 118556	*Pinus radiata*	South Africa	DQ243791	NA	NA	NA	NA
*C. leucosperma*	CFCC 89622	*Pyrus bretschneideri*	Gansu, China	KR045616	KR045698	KU710988	KU710944	KU710911
*C. leucosperma*	CFCC 89894	*Pyrus bretschneideri*	Qinghai, China	KR045617	KR045699	KU710989	KU710945	KU710912
*C. leucostoma*	CFCC 53140	*Prunus sibirica*	Beijing, China	MN854445	MN854656	MN850760	MN850746	MN850753
*C. leucostoma*	CFCC 53141	*Prunus sibirica*	Beijing, China	MN854446	MN854657	MN850761	MN850747	MN850754
*C. longiostiolata*	MFLUCC 16-0628^T^	*Salix* × *fragilis*	Russia	KY417734	KY417768	KY417700	KY417802	NA
*C. longispora*	CBS 144236^T^	*Prunus domestica*	California, USA	MG971905	NA	MG972054	NA	MG971615
*C. lumnitzericola*	MFLUCC 17-0508^T^	*Lumnitzera racernosa*	Tailand	MG975778	MH253461	MH253457	MH253453	NA
*C. macropycnidia*	Kern907	*Vitis vinifera*	USA	OP038094	OP076935	OP003977	OP095265	OP106954
*C. mali*	CFCC 50030	*Malus pumila*	Shanxi, China	MH933643	MH933677	MH933550	MH933608	MH933524
*C. mali*	CFCC 50031	*Crataegus* sp*.*	Shanxi, China	KR045636	KR045716	KU711004	KU710965	KU710927
*C. mali-spectabilis*	CFCC 53181^T^	*Malus spectabilis ‘Royalty’*	Xinjiang, China	MK673066	MK673096	MK673036	MK673006	MK672953
*C. mali-sylvestris*	MFLUCC 16-0638	*Malus sylvestris*	Russia	KY885017	KY885018	KY885019	KY885020	NA
*C. melastoma*	A 846	*Malus domestica*	USA	AF191184				
*C. melnikii*	MFLUCC 16-0635	*Populus nigra* var. *italica*	Russia	KY417736	KY417770	KY417702	KY417804	NA
*C. melnikii*	MFLUCC 15-0851^T^	*Malus domestica*	Russia	KY417735	KY417769	KY417701	KY417803	NA
*C. mougeotii*	ATCC 44994	*Picea abies*	Norway	AY347329	NA	NA	NA	NA
*C. multicollis*	CBS 105.89^T^	*Quercus ilex* subsp. *rotundifolia*	Spain	DQ243803	NA	NA	NA	NA
*C. myrtagena*	CBS 116843^T^	*Tibouchiina urvilleana*	USA	AY347363	NA	NA	NA	NA
*C. nitschkii*	CMW 10180^T^	*Eucalyptus globulus*	Ethiopia	AY347356	NA	NA	NA	NA
*C. nivea*	MFLUCC 15-0860	*Salix acutifolia*	Russia	KY417737	KY417771	KY417703	KY417805	NA
*C. nivea*	CFCC 89641	*Elaeagnus angustifolia*	Ningxia, China	KF765683	KF765699	KU711006	KU710967	KU710929
*C. notastroma*	NE_TFR5	*Populus tremuloides*	USA	JX438632	NA	NA	NA	JX438543
*C. notastroma*	NE_TFR8	*Populus tremuloides*	USA	JX438633	NA	NA	NA	JX438542
*C. ochracea*	CFCC 53164^T^	*Cotoneaster* sp.	Xinjiang, China	MK673060	MK673090	MK673030	MK673001	MK672949
*C. oleicola*	CBS 144248^T^	*Olea europaea*	California, USA	MG971944	NA	MG972098	NA	MG971660
*C. olivacea*	CFCC 53176^T^	*Sorbus tianschanica*	Xinjiang, China	MK673068	MK673098	MK673038	MK673008	MK672955
*C. olivacea*	CFCC 53177	*Prunus virginiana*	Xinjiang, China	MK673071	MK673101	MK673041	MK673011	NA
*C. olivarum*	CBS 145585^T^	*Olea europaea*	California, USA	MK514094	NA	MK509030	NA	MK509025
*C. palm*	CXY 1276	*Cotinus coggygria*	Beijing, China	JN402990	NA	NA	NA	KJ781296
*C. palm*	CXY 1280^T^	*Cotinus coggygria*	Beijing, China	JN411939	NA	NA	NA	KJ781297
*C. paracinnamomea*	CFCC 55453^T^	*Salix matsudana*	Gansu, China	MZ702594	NA	OK303456	OK303515	OK303576
*C. paracinnamomea*	CFCC 55454	*Salix matsudana*	Gansu, China	MZ702597	NA	OK303459	OK303518	OK303579
*C. parakantschavelii*	MFLUCC 15-0857^T^	*Populus* × *sibirica*	Russia	KY417738	KY417772	KY417704	KY417806	NA
*C. parapersoonii*	T28.1^T^	*Prunus persica*	USA	AF191181	NA	NA	NA	NA
*C. parapistaciae*	CBS 144506^T^	*Pistacia vera*	California, USA	MG971804	NA	MG971954	NA	MG971519
*C. paraplurivora*	FDS-564^T^	*Prunus persica* var. *nucipersica*	Canada	OL640183	OL640185	OL631587	NA	OL631590
*C. paraplurivora*	FDS-623	*Prunus persica var. persica*	Canada	OL640181	OL640123	OL631588	NA	OL631591
*C. parasitica*	MFLUCC 15-0507^T^	*Malus domestica*	Russia	KY417740	KY417774	KY417706	KY417808	NA
*C. parasitica*	XJAU 2542-1	*Malus* sp.	Xinjiang, China	MH798884	MH798897	NA	NA	MH813452
*C. paratranslucens*	MFLUCC 15-0506^T^	*Populus alba* var.* bolleana*	Russia	KY417741	KY417775	KY417707	KY417809	NA
*C. paratranslucens*	MFLUCC 16-0627	*Populus alba*	Russia	KY417742	KY417776	KY417708	KY417810	NA
*C. pavettae*	CBS 145562^T^	*Pavetta revoluta*	South Africa	MK876386	MK876427	MK876457	MK876483	MK876497
*C. phialidica*	MFLU 16-2442^T^	*Alnus glutinosa*	Italy	MT177932	MT177959	NA	MT432209	MT454014
*C. phitsanulokensis*	MFLUCC 21-0046^T^	unidentified decaying leaves	Phitsanulok, Thailand	MZ356517	MZ356521	MZ451160	MZ451168	MZ451164
*C. piceae*	CFCC 52841^T^	*Picea crassifolia*	Xinjiang, China	MH820398	MH820391	MH820406	MH820395	MH820402
*C. piceae*	CFCC 52842	*Picea crassifolia*	Xinjiang, China	MH820399	MH820392	MH820407	MH820396	MH820403
*C. pingbianensis*	MFLUCC 18-1204^T^	*Undefined wood*	Yunnan, China	MK912135	MK571763	MN685817	NA	NA
*C. pini*	CBS 197.42	*Pinus sylvestris*	Switzerland	AY347332	NA	NA	NA	NA
*C. pini*	CBS 224.52^T^	*Pinus strobus*	USA	AY347316	NA	NA	NA	NA
*C. pistaciae*	CBS 144238^T^	*Pistacia vera*	California, USA	MG971802	NA	MG971952	NA	MG971517
*C. platanicola*	MFLU 17-0327	*Platanus hybrida*	Italy	MH253451	MH253452	MH253449	MH253450	NA
*C. platycladi*	CFCC 50504^T^	*Platycladus orientalis*	Yunnan, China	MH933645	MH933679	MH933552	MH933610	MH933516
*C. platycladi*	CFCC 50505	*Platycladus orientalis*	Yunnan, China	MH933646	MH933680	MH933553	MH933611	MH933517
*C. platycladicola*	CFCC 50038^T^	*Platycladus orientalis*	Gansu, China	KT222840	MH933682	MH933555	MH933613	MH933519
*C. platycladicola*	CFCC 50039	*Platycladus orientalis*	Gansu, China	KR045642	KR045721	KU711008	KU710973	KU710931
*C. plurivora*	CBS 144239^T^	*Olea europaea*	California, USA	MG971861	NA	MG972010	NA	MG971572
*C. populi*	CFCC 55472^T^	*Populus* sp.	Gansu, China	MZ702609	NA	OK303471	OK303530	OK303591
*C. populi*	CFCC 55473	*Populus* sp	Gansu, China	MZ702610		OK303472	OK303531	OK303592
*C. populicola*	CBS 144240^T^	*Populus deltoides*	California, USA	MG971891	NA	MG972040	NA	MG971601
*C. populina*	CFCC 89644^T^	*Salix psammophila*	Shaanxi, China	KF765686	KF765702	KU711007	KU710969	KU710930
*C. populinopsis*	CFCC 50032^T^	*Sorbus aucuparia*	Ningxia, China	MH933648	MH933683	MH933556	MH933614	MH933520
*C. predappioensis*	MFLUCC 17-2458^T^	*Platanus hybrida*	Italy	MG873484	MG873480	NA	NA	NA
*C. pruinopsis*	CFCC 50034^T^	*Ulmus pumila*	Shaanxi, China	KP281259	KP310806	KP310836	KU710970	KP310849
*C. pruinopsis*	CFCC 50035	*Ulmus pumila*	Jilin, China	KP281260	KP310807	KP310837	KU710971	KP310850
*C. pruinosa*	CBS 201.42^T^	*Syringa* sp.	Switzerland	DQ243801	NA	NA	NA	NA
*C. pruinosa*	CFCC 50036	*Syringa oblata*	Qinghai, China	KP310800	KP310802	KP310832	NA	KP310845
*C. prunicola*	MFLU 17-0995^T^	*Prunus* sp.	Italy	MG742350	MG742351	MG742353	MG742352	NA
*C. pruni-mume*	CFCC 53179	*Prunus armeniaca*	Xinjiang, China	MK673057	MK673087	MK673027	NA	MK672947
*C. pruni-mume*	CFCC 53180^T^	*Prunus mume*	Xinjiang, China	MK673067	MK673097	MK673037	MK673007	MK672954
*C. pubescentis*	MFLUCC 18-1201^T^	*Quercus pubescens*	Forlì-Cesena, Italy	MK912130	MK571758	MN685812	NA	NA
*C. punicae*	CBS 144244	*Punica granatum*	California, USA	MG971943	NA	MG972091	NA	MG971654
*C. quercicola*	MFLU 17-0881	*Quercus* sp.	Italy	MF190128	MF190074	NA	NA	NA
*C. quercicola*	MFLUCC 14-0867^T^	*Quercus* sp.	Italy	MF190129	MF190073	NA	NA	NA
*C. quercinum*	CFCC 53133^T^	*Quercus mongolica*	China	MT360045	MT360033	MT363982	MT363991	MT364001
*C. quercinum*	CFCC 53132	*Quercus mongolica*	China	MT360044	MT360032	MT363981	MT363990	MT364000
*C. rhizophorae*	MUCC302	*Eucalyptus grandis*	Australia	EU301057	NA	NA	NA	NA
*C. ribis*	CFCC 50026	*Ulmus pumila*	Qinghai, China	KP281267	KP310813	KP310843	KU710972	KP310856
*C. ribis*	CFCC 50027	*Ulmus pumila*	Qinghai, China	KP281268	KP310814	KP310844	NA	KP310857
*C. rosae*	MFLU 17-0885	*Rosa canina*	Italy	MF190131	MF190076	NA	NA	NA
*C. rosicola*	CF 20197024^T^	*Rosa* sp.	Tibet, China	MK673079	MK673109	MK673049	MK673019	MK672965
*C. rosigena*	MFLUCC 18-0921^T^	*Rosa* sp.	Russia	MN879872	MN879873	NA	NA	NA
*C. rostrata*	CFCC 89909^T^	*Salix cupularis*	Gansu, China	KR045643	KR045722	KU711009	KU710974	KU710932
*C. rostrata*	CFCC 89910	*Salix cupularis*	Gansu, China	KR045644	KR045723	KU711010	KU710975	KU710933
*C. rusanovii*	MFLUCC 15-0854^T^	*Salix babylonica*	Russia	KY417744	KY417778	KY417710	KY417812	NA
*C. salicacearum*	MFLUCC 15-0861	*Salix* × *fragilis*	Russia	KY417745	KY417779	KY417711	KY417813	NA
*C. salicacearum*	MFLUCC 15-0509^T^	*Salix alba*	Russia	KY417746	KY417780	KY417712	KY417814	NA
*C. salicicola*	MFLUCC 14-1052^T^	*Salix alba*	Russia	KU982636	KU982635	KU982637	NA	NA
*C. salicina*	MFLUCC 15-0862^T^	*Salix alba*	Russia	KY417750	KY417784	KY417716	KY417818	NA
*C. salicina*	MFLUCC 16-0637	*Salix* × *fragilis*	Russia	KY417751	KY417785	KY417717	KY417819	NA
*C. salicis-albae*	MFLUCC 18-0485	*Salix alba*	Russia	MT734820	MT734819	OL754585	OL754584	NA
*C. schulzeri*	CFCC 50040	*Malus domestica*	Ningxia, China	KR045649	KR045728	KU711013	KU710980	KU710936
*C. schulzeri*	CFCC 50042	*Malus pumila*	Gansu, China	KR045650	KR045729	KU711014	KU710981	KU710937
*C. shoreae*	MFLUCC 21-0047^T^	*Shorea* sp.	Chiang Mai,Thailand	MZ356515	MZ356519	MZ451158	MZ451166	MZ451162
*C. shoreae*	MFLUCC 21-0048	*Shorea* sp.	Chiang Mai,Thailand	MZ356516	MZ356516	MZ356516	MZ356516	MZ356516
*C. sibiraeae*	CFCC 50045^T^	*Sibiraea angustata*	Gansu, China	KR045651	KR045730	KU711015	KU710982	KU710938
*C. sibiraeae*	CFCC 50046	*Sibiraea angustata*	Gansu, China	KR045652	KR045731	KU711015	KU710983	KU710939
*C. sophorae*	CFCC 50048	*Magnolia grandiflora*	Shanxi, China	MH820401	MH820394	MH820409	MH820397	MH820405
*C. sophorae*	CFCC 89598	*Styphnolobium japonicum*	Gansu, China	KR045654	KR045733	KU711018	KU710985	KU710941
*C. sophoricola*	CFCC 89596	*Styphnolobium japonicum* var*. pendula*	Gansu, China	KR045656	KR045735	KU711020	KU710987	KU710943
*C. sophoricola*	CFCC 89595^T^	*Styphnolobium japonicum* var*. pendula*	Gansu, China	KR045655	KR045734	KU711019	KU710986	KU710942
*C. sophoriopsis*	CFCC 89600^T^	*Styphnolobium japonicum*	Gansu, China	KR045623	KP310804	KU710992	KU710951	KU710915
*C. sorbi*	MFLUCC 16-0631^T^	*Sorbus aucuparia*	Russia	KY417752	KY417786	KY417718	KY417820	NA
*C. sorbicola*	MFLUCC 16-0584^T^	*Acer pseudoplatanus*	Russia	KY417755	KY417789	KY417721	KY417823	NA
*C. sorbicola*	MFLUCC 16-0633	*Cotoneaster melanocarpus*	Russia	KY417758	KY417792	KY417724	KY417826	NA
*C. sorbina*	CF 20197660^T^	*Sorbus tianschanica*	Xinjiang, China	MK673052	MK673082	MK673022	NA	MK672943
*C. spiraeae*	CFCC 50049^T^	*Spiraea salicifolia*	Gansu, China	MG707859	MG707643	MG708196	MG708199	NA
*C. spiraeae*	CFCC 50050	*Spiraea salicifolia*	Gansu, China	MG707860	MG707644	MG708197	MG708200	NA
*C. spiraeicola*	CFCC 53138^T^	*Spiraea salicifolia*	Beijing, China	MN854448	MN854659	NA	MN850749	MN850756
*C. spiraeicola*	CFCC 53139	*Tilia nobilis*	Beijing, China	MN854449	MN854660	NA	MN850750	MN850757
*C. tamaricicola*	CFCC 50508^T^	*Tamarix chinensis*	Yunnan, China	MH933652	MH933687	MH933560	MH933617	MH933523
*C. tanaitica*	MFLUCC 14-1057^T^	*Betula pubescens*	Russia	KT459411	KT459412	KT459413	NA	NA
*C. thailandica*	MFLUCC 17-0262^T^	*Xylocarpus moluccensis*	Thailand	MG975776	MH253463	MH253459	MH253455	NA
*C. thailandica*	MFLUCC 17-0263^T^	*Xylocarpus moluccensis*	Thailand	MG975777	MH253464	MH253460	MH253456	NA
*C. tibetensis*	CF 20197026	*Cotoneaster* sp.	Tibet, China	MK673076	MK673106	MK673046	MK673016	MK672962
*C. tibetensis*	CF 20197032^T^	*Cotoneaster* sp.	Tibet, China	MK673078	MK673108	MK673048	MK673018	MK672964
*C. tibouchinae*	CPC 26333^T^	*Tibouchina semidecandra*	France	KX228284	KX228335	NA	NA	NA
*C. translucens*	CXY 1351	*Populus davidiana*	Inner Mongolia, China	KM034874	NA	NA	NA	NA
*C. ulmi*	MFLUCC 15-0863^T^	*Ulmus minor*	Russia	KY417759	NA	NA	NA	NA
*C. ulmicola*	MFLUCC 18-1227^T^	*Ulmus pumila*	Russia	MH940220	MH940218	MH940216	NA	NA
*C. unilocularis*	MFLUCC 15-0481^T^	*Tamarix* sp.	Italy	KU900332	KU900304	NA	KX011166	NA
*C. valsoidea*	CMW 4309^T^	*Eucalyptus grandis*	Indonesia	AF192312	NA	NA	NA	NA
*C. variostromatica*	CMW 6766^T^	*Eucalyptus globulus*	Australia	AY347366	NA	NA	NA	NA
*C. variostromatica*	CMW 1240	*Eucalyptus grandis*	South Africa	AF260263	NA	NA	NA	NA
*C. verrucosa*	CFCC 53157^T^	*Platycladus orientalis*	Beijing, China	MW418408	MW418420	NA	MW422911	MW422923
*C. verrucosa*	CFCC 53158	*Platycladus orientalis*	Beijing, China	MW418410	MW418422	MW422901	MW422913	MW422925
*C. vinacea*	CBS 141585^T^	*Vitis interspecific* hybrid ‘Vidal’	USA	KX256256	NA	NA	NA	KX256277
*C. viridistroma*	CBS 202.36^T^	*Cercis canadensis Castigl*	USA	MN172408	MN172388	NA	NA	MN271853
*C. viticola*	Cyt2	*Vitis interspecific* hybrid ‘Frontenac’	USA	KX256238	NA	NA	NA	KX256259
*C. viticola*	CBS 141586^T^	*Vitis vinifera ‘*Cabernet Franc’	USA	KX256239	NA	NA	NA	KX256260
*C. xinglongensis*	CFCC 52458	*Castanea mollissima*	China	MK432622	MK429892	MK442946	MK578082	NA
*C. xinglongensis*	CFCC 52459	*Castanea mollissima*	China	MK432623	MK429893	MK442947	MK578083	NA
*C. xinjiangensis*	CFCC 53182	*Rosa* sp.	Xinjiang, China	MK673064	MK673094	MK673034	MK673004	MK672951
*C. xinjiangensis*	CFCC 53183^T^	*Rosa* sp.	Xinjiang, China	MK673065	MK673095	MK673035	MK673005	MK672952
*C. xylocarpi*	MFLUCC 17-0251^T^	*Xylocarpus granatum*	Thailand	MG975775	MH253462	MH253458	NA	NA
*C. yakimana*	Bent902/CBS 149297	*Vitis vinifera*	USA	OM976602	ON059350	ON012555	ON045093	ON012569
*C. yakimana*	Bent903/CBS 149298	*Vitis vinifera*	USA	OM976603	ON059351	ON012556	ON045094	ON012570
*C. zhaitangensis*	CFCC 56227^T^	*Euonymus japonicus*	China	OQ344750	NA	OQ410623	OQ398733	OQ398760
*C. zhaitangensis*	CFCC 57537	*Euonymus japonicus*	China	OQ344751	NA	OQ410624	OQ398734	OQ398761
*Diaporthe eres*	CBS 145040	*Lactuca satia*	Netherlands	MK442579	MK442521	MK442634	MK442663	MK442693
*Diaporthe vaccinii*	CBS 160.32	*Vaccinium macrocarpon*	USA	KC343228	NA	JQ807297	NA	KC343954

^a^ATCC: American Type Culture Collection, Virginia, USA; CBS: Westerdijk Fungal Biodiversity Institute (CBS-KNAW Fungal Biodiversity Centre), Utrecht, The Netherlands; CFCC: China Forestry Culture Collection Centre, Beijing, China; CMW: Culture collection of Michael Wingfield, University of Pretoria, South Africa; CPC: Culture collection of Pedro Crous, The Netherlands; IMI: Culture collection of the International Mycological Institute, CABI Bioscience, Egham, Surrey, UK; MFLU: Mae Fah Luang University herbarium, Thailand; MFLUCC: Mae Fah Luang University Culture Collection, Thailand; MUCC: Murdoch University Culture Collection, Perth, Australia; NE: Gerard Adams collections, University of Nebraska, Lincoln NE, USA; XJAU: Xinjiang Agricultural University, Xinjiang, China; IRAN: the Fungal Culture Collection of the Iranian Research Institute of Plant Protection; FCCUU: Fungal Culture Collection of Urmia University. NA: not applicable. All the new isolates used in this study are in bold and the type materials are marked with ^T^.

The newly generated sequences were checked and trimmed manually in BioEdit v. 7.2.6^70^ and deposited in GenBank (Table 3). Sequences based on the combined dataset (ITS-rDNA, LSU, *act1*, *rpb2*, and *tef1-α*) were aligned using the MAFFT v. 7 online service (https://mafft.cbrc.jp/alignment/server/)^71^ for each locus separately by including the sequences of ex-type and representative *Cytospora* strains available in the literature and adjusted where necessary. The concatenated sequence dataset (ITS-rDNA, LSU, *act1*, *rpb2*, and *tef1-α*) was produced in Mesquite v. 2.74^72^ and used for phylogenetic analysis. Multi-gene phylogenetic analyses were done by using Maximum Likelihood (ML), Maximum Parsimony (MP), and Bayesian Inference (BI) methods. ML analysis was conducted in RAxML-HPC BlackBox v. 8.2.12^73^ provided by the CIPRES Science Gateway v 3.3^74^. The substitution model was set as GTRGAMMA+I and branch stability was estimated by 1000 bootstrap replications to produce a cladogram with nodal support values. BI was performed in MrBayes v. 3.2.7^75^ by using the Markov Chain Monte Carlo (MCMC) method with four chains, 1M generations, and a temperature value of the heated chain of 0.1. Trees were saved every 1000 generations, Burn-in was set to 25%, and posterior probabilities (PP) were determined from the remaining trees. For determining the best-fit evolutionary models required for BI, all individual alignments were evaluated in MrModeltest v2.3^76^ using the Akaike Information Criterion (AIC). MP analysis was performed in PAUP (Phylogenetic Analysis Using Parsimony) v. 4.0b10^77^. Trees were inferred using the heuristic search option with 1000 random sequence additions and branch swapping with the tree-bisection-reconnection (TBR) algorithm and gaps were treated as missing data. The bootstrap values with 1000 replicates were performed to determine branch support. Descriptive tree statistics [Tree Length (TL), Consistency Index (CI), Retention Index (RI), and Homoplasy Index (HI)] were calculated for trees generated in the parsimony analysis. Sequences of *Diaporthe eres* CBS 145040 and *Diaporthe vaccinii* CBS 160.32 were used as outgroups. The resultant phylogenetic tree was visualized in FigTree v. 1.4.4^78^ and edited in graphic design software, Adobe Illustrator CC 2018 (Adobe Inc., San Jose, California). The ultimate concatenated alignment and ML-generated tree file were submitted to TreeBASE (https://www.treebase.org) under the accession number 29113. Sequence data were deposited in the GenBank dataset and their accession numbers are provided in Table 3.

### Morphological characterization

Purified cultures were grown on PDA medium, incubated in the dark at 25 ± 1 °C, and examined after three, seven, and 30 days. Radial growth was measured by taking two measurements perpendicular to each other in triplicates^21,57,60,79^. Colony color was determined based on Rayner’s color charts^80^. Pycnidia formation was induced on pine needles embedded in 2% water agar [20 g Agar (Merck, Darmstadt, Germany) in 1000 mL distilled water] medium or on one-year-old apple shoots embedded in PDA medium and incubated under near ultraviolet (NUV) light (12 h photoperiod) at room temperature. Both pine needles and apple shoots were autoclave sterilized at 121 °C for 20 min. thrice, with a 24-h interval between each sterilization. Pycnidia formation was checked weekly for 30 days. Hand sections of the conidiomata (both transverse and longitudinal) were prepared and mounted in water or lactic acid and examined for morphological details. Macro-morphological characters including size and arrangement of stromata, presence or absence of conceptacle, number, and diameter of ostioles per ectostromatic disk, arrangement of locules and color, shape, and size of discs were examined using an Olympus SZX-ILLB200 dissecting microscope. Micro-morphological characters including the shape and size of conidia (n = 50) and conidiophores/conidiogenous cells (n = 25) were determined at 1000× magnification under an Olympus AX70 compound microscope with differential interference contrast (DIC) illumination. Adobe Photoshop 2020 v. 2.10.8 software (Adobe Inc., San Jose, California) was used for manual editing.

### Pathogenicity trials

Pathogenicity trials were done based on the standard and routine method described in the literature^31,42,44,57,81–84^. Detached, dormant, one- or two-year-old, 25 × 1.5–2 cm apple shoots of the cv. ʻRed Delicious’ were collected from healthy trees in the apple cultivar collection farm of Urmia University. The shoots were washed under running tap water, surface disinfested with 75% ethanol for 4 min., washed again in sterile distilled water, and blotted dry on a sterile paper towel. The bark of the shoots was removed in the center with a 5-mm-diameter flame sterilized cork borer and inoculated with a 5 mm diameter mycelial plug of actively growing fungal isolates (7–day-old on PDA). All the obtained isolates were used in pathogenicity trials. Each inoculated site was covered with a sterile moistened cotton ball and wrapped with Parafilm™ (Bemis™, pm996, USA) to maintain the moisture. Sterile PDA plugs were used as the controls. Six shoots were used for each fungal isolate and control treatment. Inoculated shoots were placed in clean plastic containers containing three layered moistened sterile paper towels and incubated under laboratory conditions (diurnal light, 25 ± 2 °C, 80% relative humidity) for 21 days. All the experiments were repeated once under similar conditions. Length of bark and wood discoloration around the inoculated sites were measured 21 days post-inoculation. Also, the pathogenicity of the most virulent isolate (BA 2-4) was determined on the ‘Red Delicious’ cultivar under field conditions. Four 2–3-year-old branches in four geographical directions were selected. The bark of the branches was surface disinfected by spraying with 75% ethanol, and the fungal inoculation was the same as described for detached shoots. Inoculation was done on April 7, 2023, and the results were evaluated on May 24, 2023.

In addition, five fungal isolates (BA 1-1, BA 2-1, BA 2-4, KU 1-1, and BA 3-1 isolates) which had the highest virulence in the trials as mentioned above (Table 1) were chosen for the evaluation of reaction of 12 apple cultivars including ʻBraeburn’, ʻDelbard Estivale’, ʻFuji’, ʻGranny Smith’, ʻGolden Delicious’, ʻGolden Primrose’, ʻIdared’, ʻRed Delicious’, ʻM4’, ʻM7’, ʻMM106’ and ʻMM109’ against these isolates. Healthy shoots were collected from the apple cultivar collection farm of Urmia University and used for pathogenicity tests as described above and the length of bark and wood discoloration around the inoculated sites was measured 21 days post-inoculation. Experiments were laid down following a completely randomized design (CRD). The pathogenicity data were transformed by square root due to the existence of zero values and were subjected to analysis of variance (ANOVA) using SAS v.9.1 software (SAS Institute, Inc., USA). The lesion length means were compared with Duncan’s multiple range test (*P* ≤ 0.05). To confirm Koch’s postulates, fungal re-isolation was carried out from the margins of the developed lesions in all symptomatic samples, and isolates were re-identified morphologically as described previously.

### Supplementary Information

Supplementary Figure S1.

## Data Availability

All sequence data generated in this study are available in NCBI GenBank (https://www.ncbi.nlm.nih.gov/genbank/) following the accession numbers MZ948960-MZ948962 (ITS); MZ948957-MZ948959 (LSU); MZ997842-MZ997844 (*act1*); MZ997845-MZ997847 (*rpb2*) and MZ997848-MZ997850 (*tef1α*). Also, the ultimate concatenated alignment and ML-generated tree file were submitted to TreeBASE (https://www.treebase.org) under the accession number 29113. All data analyzed during this study are included in this manuscript.
