# Supplementary figures and images for: Apple crown and collar canker and necrosis caused by Cytospora balanejica sp. nov. in Iran
Source: Sci Rep. 2024 Mar 19;14:6629. doi: 10.1038/s41598-024-57235-3 (PMC10951349; doi:10.1038/s41598-024-57235-3)

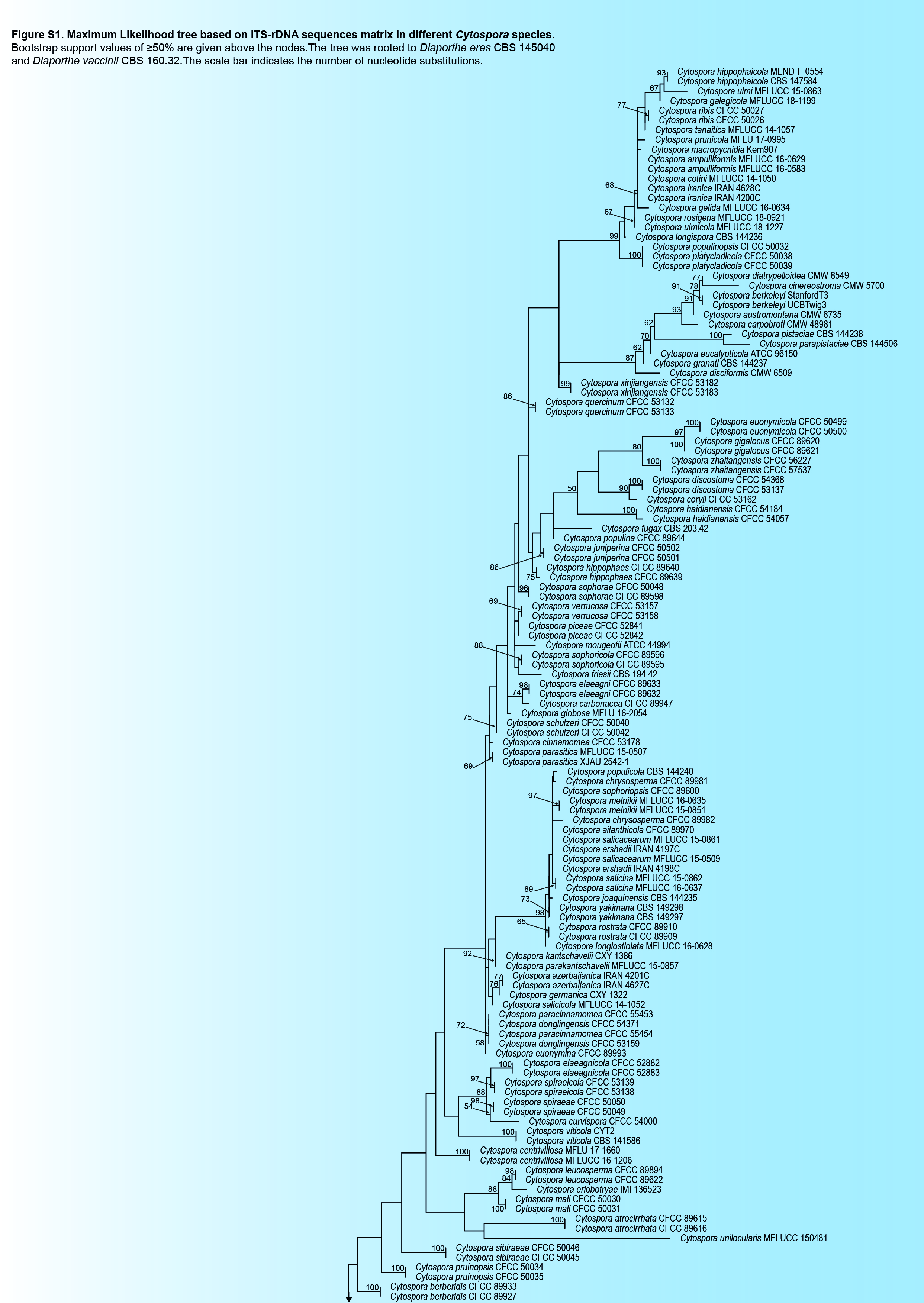

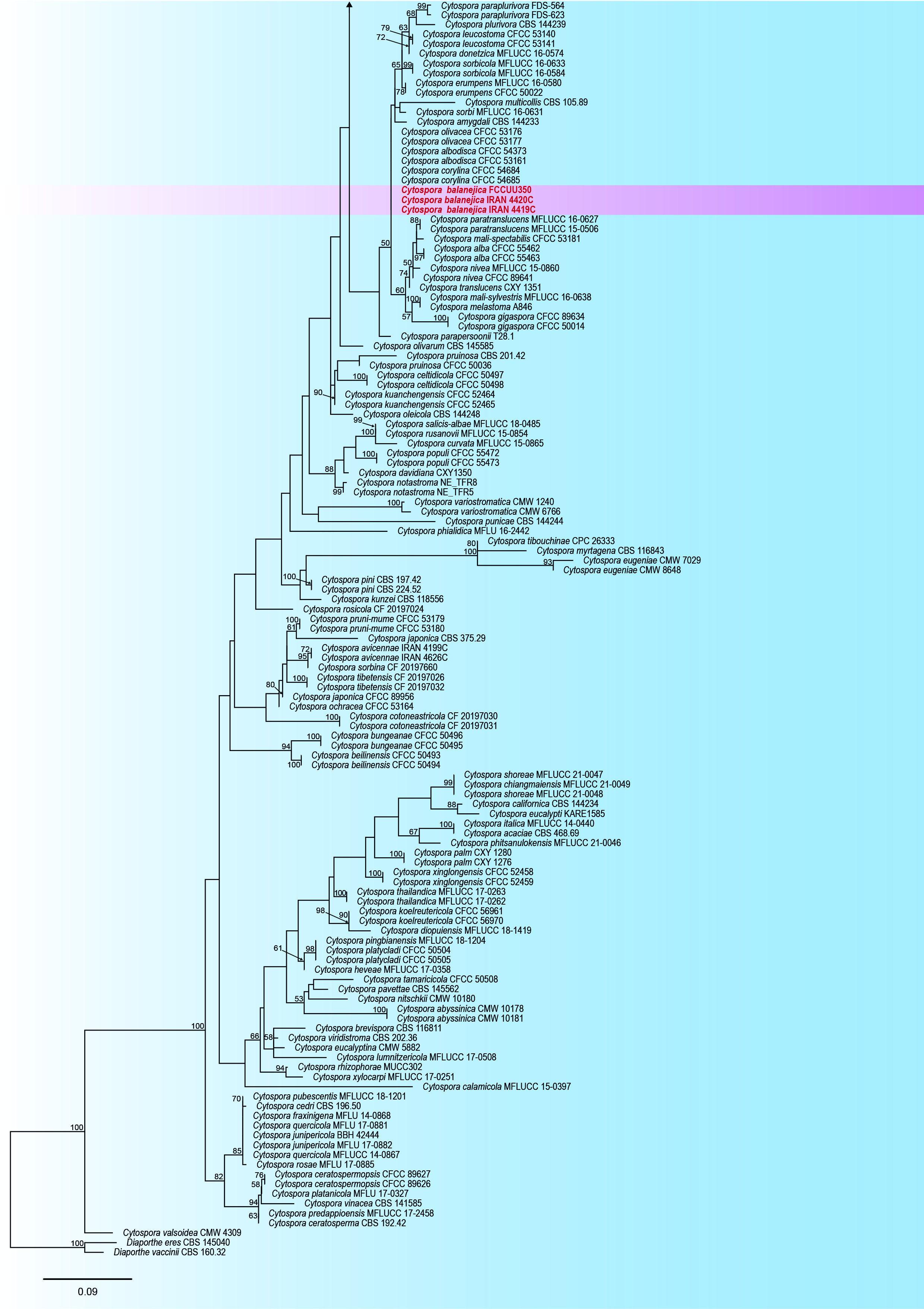

Supplement: Supplementary file 1 — Supplementary Figure S1. [file 41598_2024_57235_MOESM1_ESM.docx]
